# Mapping Muscles Activation to Force Perception during Unloading

**DOI:** 10.1371/journal.pone.0152552

**Published:** 2016-03-31

**Authors:** Simone Toma, Francesco Lacquaniti

**Affiliations:** 1 Centre of Space Bio-medicine, University of Rome Tor Vergata, Rome, Italy; 2 Laboratory of Neuromotor Physiology, IRCCS Santa Lucia Foundation, Rome, Italy; 3 Department of Systems Medicine, University of Rome, Tor Vergata, Rome, Italy; Shanghai Jiao Tong University, CHINA

## Abstract

It has been largely proved that while judging a force humans mainly rely on the motor commands produced to interact with that force (i.e., sense of effort). Despite of a large bulk of previous investigations interested in understanding the contributions of the descending and ascending signals in force perception, very few attempts have been made to link a measure of neural output (i.e., EMG) to the psychophysical performance. Indeed, the amount of correlation between EMG activity and perceptual decisions can be interpreted as an estimate of the contribution of central signals involved in the sensation of force. In this study we investigated this correlation by measuring the muscular activity of eight arm muscles while participants performed a quasi-isometric force detection task. Here we showed a method to quantitatively describe muscular activity (“*muscle-metric function”*) that was directly comparable to the description of the participants' psychophysical decisions about the stimulus force. We observed that under our experimental conditions, muscle-metric absolute thresholds and the shape of the muscle-metric curves were closely related to those provided by the psychophysics. In fact a global measure of the muscles considered was able to predict approximately 60% of the perceptual decisions total variance. Moreover the inter-subjects differences in psychophysical sensitivity showed high correlation with both participants' muscles sensitivity and participants' joint torques. Overall, our findings gave insights into both the role played by the corticospinal motor commands while performing a force detection task and the influence of the gravitational muscular torque on the estimation of vertical forces.

## Introduction

To carry out most of our everyday actions, both internal forces—e.g., muscular torques as well as external forces—e.g., gravity—must be taken into account. For instance, the simple action of maintaining the arm in a fixed posture without external support entails an active muscular torque to resist the downward gravitational force acting on the arm. Moreover, if an additional external force is applied to the arm while in the same posture, our percept of the external force will result from the combination of two kinds of signals: one derived from the descending motor commands necessary to counteract the external force and to maintain the posture, and the second one derived from the resulting afferent somaesthetic signals. Several different approaches, such as psychophysics, analysis of kinematics and dynamics, electromyography (EMG), and computational modeling of motor control, have been used to investigate both the mechanisms of how the central nervous system (CNS) interacts with external forces and how it merges descending and ascending signals to perform a perceptual decision.

On the one hand, psychophysical methods have been largely exploited in those studies where central and peripheral signals were decoupled during force detection or discrimination tasks. Common tasks for these studies were active and passive force/mass perception [1,2,3], torque and stiffness discrimination [4], force perception during voluntary and induced muscle contractions [5,6], perception of force under fatigued and non-fatigued muscles conditions [7,8,9], force discrimination of healthy and de-afferented subjects [10,11,12,13], participants under anaesthetized hand conditions [14,15,16], as well as perception of muscular effort involving different groups of muscles [17]. The common observation of these works of a perceptual sensitivity reduction when efferent signals were manipulated gave behavioral support to the idea that force perception is mainly mediated by central signals [5,16,18,19].

On the other hand relevant insights into the mechanisms underpinning motor organization in response to external dynamic changes came from behavioral studies where either a force perturbation (i.e., force field) or a load was applied to the moving arm [20]. In these investigations either motor errors (see [21,22] for a review) or EMG activity [23] were analyzed. Among these studies, Thoroughman et al. [24] designed an elegant force field protocol where both motor errors and EMG changes were recorded simultaneously. These authors showed that descending motor commands associated with changes in EMG signals correlate with the formation of an internal representation of dynamics.

The aim of our study was to provide a measure of neural output (i.e., EMG) in relation to psychophysical performance (i.e., sense of muscular effort) to give insights into the neural basis of force perception. Indeed, the amount of correlation between EMG activity and perceptual decisions can be interpreted as an estimate of the contribution of central signals involved in force perception. Differently from one of the few studies quantifying [11,12] the correlation between muscles activity and subjects’ perceived muscular effort that provided an index of dis/concordance, we show a method to quantitatively describe muscular activity (“*muscle-metric function”*) that was directly comparable to the description of psychophysical decisions in a force detection task.

Inspired by the concepts of neuro-metrics exploring the links between neural activity and perceptual decisions (see [25] for a review), we interpreted the concomitant arm muscles activity as the result of specific central neural modulations associated with the stimulus force applied on the subject’s arm. Indeed, a body of evidence exists demonstrating a direct link between the primary motor cortex (M1) and force perception. These works, for instance, showed that transcranial magnetic brain stimulation (TMS) applied on M1 both increased participants' sense of effort [26] and attenuated improvements in the ability to accurately judge force output [27]. In the light of these previous findings, the primary aim of this study was to investigate to what extent the activity of eight arm muscles, considered as a major output of spinal alpha-motoneurons and indirectly reflecting corticospinal motor commands, were able to predict participants’ perceptual decision about force. Secondly we discussed our findings in terms of the influence of the gravitational muscular torque on the perception of vertical forces applied on arm.

## Materials and Methods

### Participants

Fourteen volunteers participated in this study (seven males and seven females, mean age = 25.4 yrs ± 5.5; mean height = 1.69 m ± 0.6; mean weight = 65.1 kg ± 8.8). All participants were right-handed (as assessed by a short questionnaire based on the Edinburgh scale), were naïve with respect to the aim of the study, and gave their written informed consent. None of them had neuromuscular disorders and all had normal or corrected-to-normal vision. The study was approved by the independent ethics committee of Fondazione Santa Lucia, Rome.

### Apparatus

**Fig 1:**
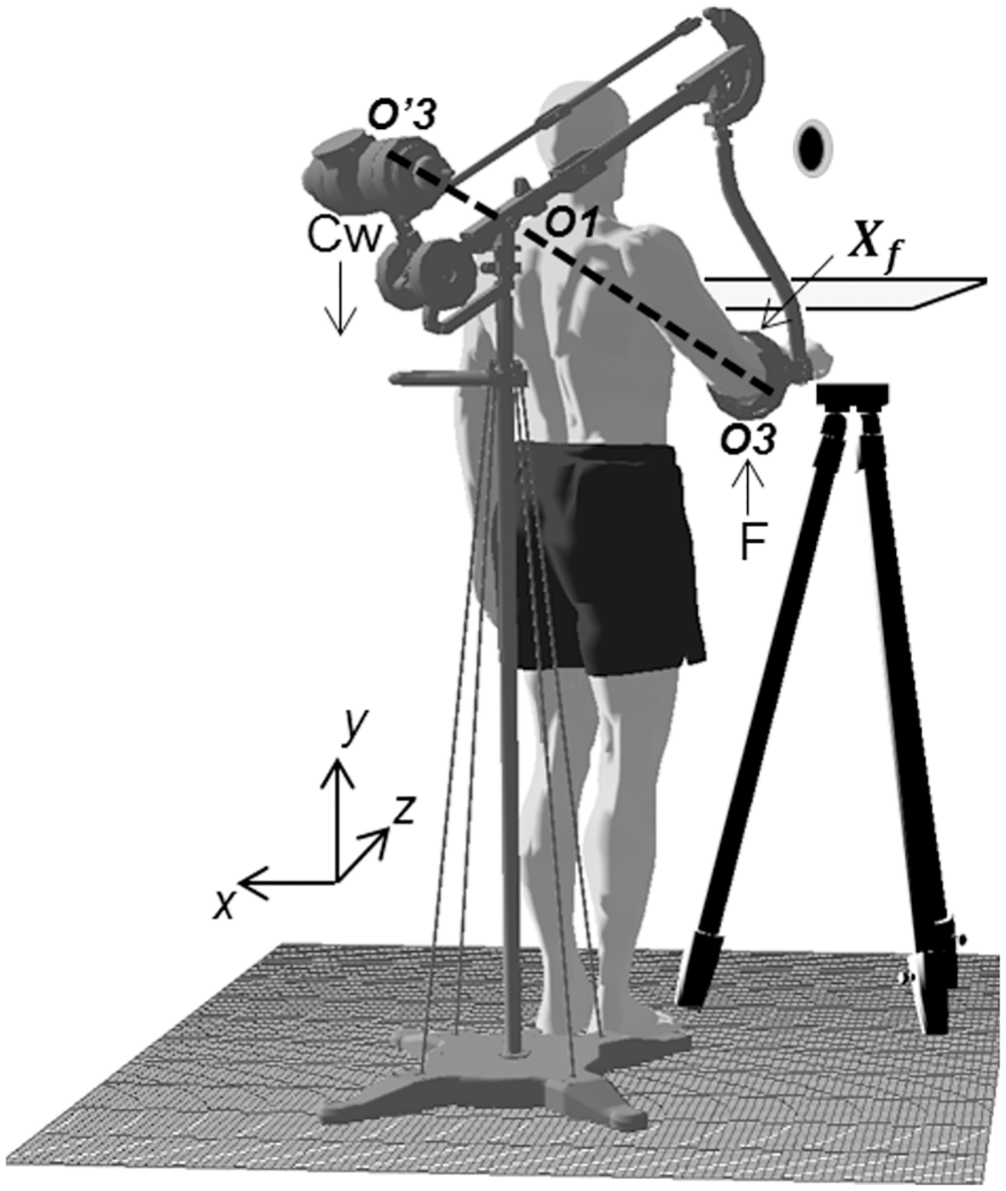
Apparatus. Trackhold (TH) device and subjects’ arm posture at the moment of force detection, namely when the black sphere (i.e. cursor) was within the gray sphere (i.e., target). *Cw* represents the load applied on the device balancer. *F* indicates the consequent upward force exerted on subjects’ arm (*F = CW* /3). *Xf* is a distance from elbow joint representing the point of force application on subjects arm. *O*3 is the contact point between subject’s arm and TH (cradle), *O1* is the fulcrum of TH horizontal lever arm and *O’*3 is the center of TH balancer. While in target, mean value of shoulder adduction angles across subjects was 8.3° ± 7°, shoulder flexion angle was 12.3° ± 5.1° and elbow elevation angles was 83.1° ± 4°.

Fig 1 shows the device we used (i.e., Track-Hold, TH, [28,29]) to track subject’s arm motion and to produce upward forces on their arm. TH is a passive device composed of a fixed base and of a movable interaction element which is adapted to be located integrally to the subject’s arm in order to permit most of the natural upper limb workspace. Moreover TH creates lever arms of its various rigid links as a result of the load (i.e., counterweight, *Cw*) applied on its balancer. In particular the downward force exerted by Cw produces an upward force *F* that is equal to Cw/3 (see paragraph 1.1 in S1 Text) at the point where the device is applied on subject’s arm, i.e., *Xf* (see Fig 1 and paragraph 1.1 in S1 Text), and it is assumed to be constant at any configuration of the device, that is at any configuration of participants’ arm. Individual TH application point *Xf* is expressed as a point along the forearm specified by a distance from the elbow joint whose mean ± SD values across subjects was 9.04 ± 0.57 cm. The counterweights applied on the TH balancer consisted of the combination of 12 brass cylinders: three samples of 1.5 N, five samples of 3 N, two samples of 10 N and two of 30 N. Thus the range of loads applied on TH balancer was from 0 to 99.5 N with a minimum step of change of about 1.5 N and a load of 1 N necessary to compensate the weight of the TH movable interaction element. Consequently the actual upward forces *F* applied on participants' arm at point *Xf* ranged from 0 to 33 N (*F* = 99 N/3) with a minimum delta of change (i.e., step size) of 0.5 N.

### Visual feedback

In order to help participants to maintain a quasi-isometric posture during upward force applications on their arm, we provided them with a 3D visual feedback of their forearm position. A target sphere (7.5 cm radius, depicted in gray in Fig 1) was placed at the center of the Virtual Reality workspace (see paragraph 1.2 in S1 Text). A cursor sphere (5.5 cm radius, black in Fig 1) represented the position of participants’ right hand, and it moved on the scene following participants’ motions (see paragraph 1.2 in S1 Text). The time delay between the real motion of the hand and its projection on the screen was about 30 msec (as determined by separate calibrations of the system). In order to make the virtual target sphere easily reachable, its distance from the subject (i.e., z axis) was adjusted with respect to the forearm length of each individual participant. Subjects’ forearm and hand were out of sight throughout the experiment, being occluded by the horizontal projection panel (Fig 1). To rule out the possibility that upper arm and TH links might be partially seen during the task, we shifted target and cursor position upward along the y-axis to display both spheres always approximately at subject’s eye height and aligned with the subject’s hand (Fig 1).

### Procedure

**Fig 2:**
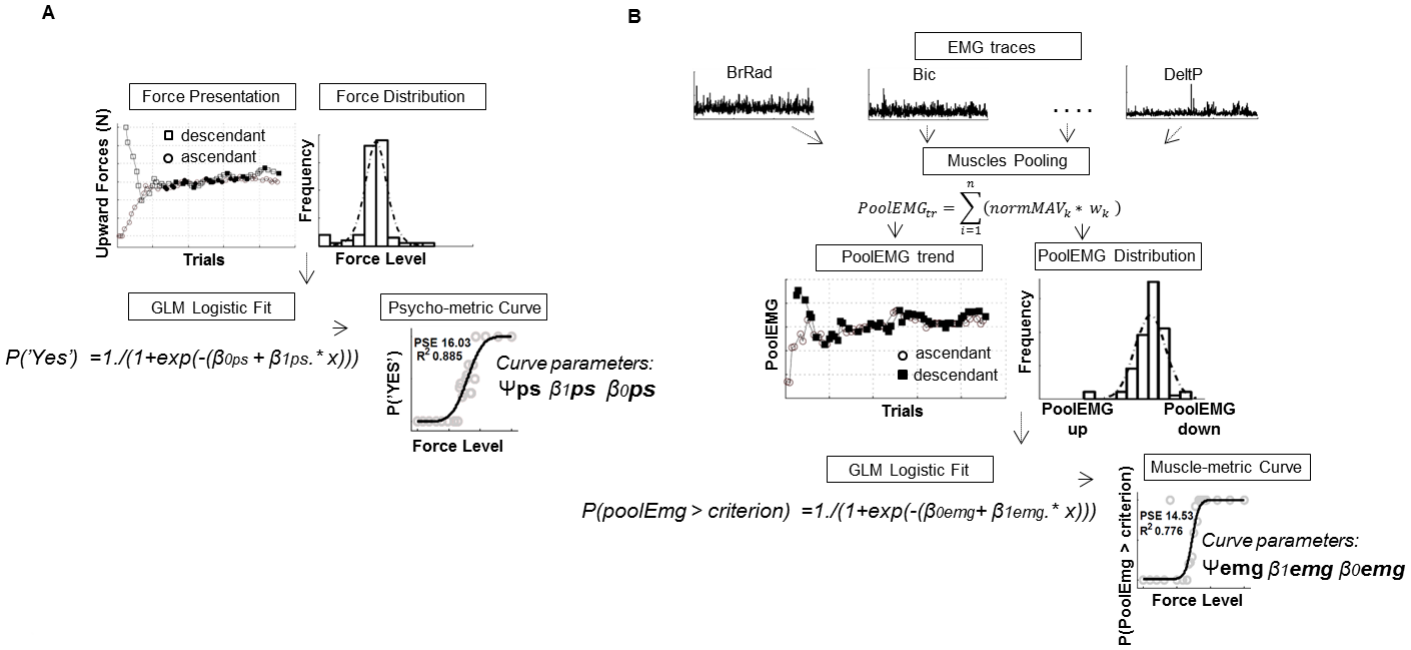
Psycho & Muscle metric curve computation from a typical subject. **A)** For each subject, the computation of the psychometric curve was obtained by extracting the distribution of the force level explored by the participant during the UP-DOWN staircase adaptive procedure and by fitting a logistic function to the probability of a positive answer with respect to each external force level (x). Ψ*ps*, *β1ps*, *β0ps* indicate the absolute threshold (PSE), the slope and the intercept, respectively, characterizing the logistic function that best explains the probability of detecting an upward force given each stimulus force. **B)** Computation of the muscle-metric curve was obtained by means of a weighted pool of all muscle activity (PoolEMG) and then fitting a logistic function to the probability of a PoolEMG value to be higher than the criterion per each external force level presented (x). Criterion level was set to the mean of the PoolEMG distribution (paragraph 1.4 in S1 Text for details). Ψ*emg*, *β1emg*, *β0emg* indicate the absolute threshold (PSE), the slope and the intercept, respectively, describing the logistic function that best explains the probability of each force level to elicit a PoolEMG value above criterion.

Participants stayed upright in front of the virtual reality system with the TH device applied on their right forearm at the pre-calculated *Xf* point (see Apparatus). Before the experiment, participants were asked to place their upper arm aligned with the trunk and forearm parallel to ground. Then, to avoid that neither the TH ring nor the TH cradle where the arm laid could touch subjects’ trunk, participants were asked to place the upper arm slightly forward (shoulder flexion angle averaged across subjects: 12.3° ± 5.1° SD) and slightly outward (shoulder adduction angles across subjects: 8.3° ± 7.0°) with respect to the trunk (Fig 1). Once in this position, subjects’ arm posture (i.e., shoulder, elbow and wrist coordinates) was stored, and both target and cursor spheres were displayed in the same position as described in the previous paragraph and depicted in Fig 1. This measure of desired arm posture at the target position was used throughout the experiment to prevent subjects from changing arm configuration across trials. Each trial started with participant's arm in a starting position that consisted in placing the hand on a tripod located in the same vertical and sagittal plane of the target, but approximately 5 cm rightward (x axis) from target position (Fig 1). With this configuration, participants were able to easily place the cursor within the target sphere just with a relative small inward motion. While participants were in the starting position, the experimenter, who was placed behind the subject, either loaded or unloaded the TH balancer with a given counterweight Cw to change the level of the upward force (*F*) exerted on subjects' arm to a new unpredictable level(see below). Once the loading/unloading operation was terminated and the new upward force level was applied on the subject’s arm, the trial was started by the experimenter and a sound informed subjects to make the small movement required to bring the cursor within the target. When the cursor was placed at the target, the cursor became green. Every time that *Xf* coordinates (roto-translated in cursor coordinates every 10 ms) deviated from the center of target sphere by more than 2 cm, the cursor turned red (60 Hz). After two seconds of permanence within the target tolerance limits, the target sphere disappeared and from that time on participants could provide their answer to the experimental question: “*do you feel an upward force acting on your arm*?”. Participants were asked to judge the experienced force only while they had the arm still at the target, without considering any force perception sensed either during the loading/unloading operation or during the small arm displacement towards the target. No time limit was imposed to the subjects to give their answer, but they had to stay within the target tolerance limits until they provided the answer. Nevertheless, subjects mostly provided their answers right after that target sphere was extinguished. Furthermore, to be sure that subjects maintained approximately the same arm configuration at each trial, we also set a tolerance motion limits of 5 cm for both the shoulder and the elbow joints in each of the three coordinates. In fact as soon as participants moved either the elbow or the shoulder more than 5 cm away from the desired arm posture set at the beginning of the experiment, the cursor sphere turned black and the answer could not be entered unless the correct configuration of shoulder and elbow joints was restored. Participants’ response was provided by pressing the keys of a response box held in their left hand relaxed along the body. A positive answer to the experimental question corresponded to a detection of an upward force, while a negative answer could correspond to the perception of any other type of force, such as a null force or a downward force. Once the answer was provided, a new sound was played to inform that the trial ended and the participant had to come back to the starting, tripod position. Force presentation followed a double (ascendant/descendant) interleaved UP-DOWN staircase [30,31] where the stimulus level at each trial was selected with respect to the answer in the previous trial. The first trial of the ascending staircase started with a zero upward force (0 N), while the descending staircase started with the maximum upward force (30 N) (see *Force Presentation* plot in Fig 2A). The upward force was increased after a negative answer and it was decreased after a positive one. This method allowed us to obtain a convergence of responses after relatively few trials (*Force Presentation* plot in Fig 2A), around a stimulus level that produced 50% of positive answers (i.e., Point of Subjective Equality, PSE). Such a force level was assumed to be the individual absolute threshold in detecting upward forces applied on the arm, that is, the stimulus intensity above and below which participants will increase the probability of giving a positive or negative answers, respectively. As suggested by previous work on vision and audition where the range of threshold values were unknown a priori [31,32], we set a large step size (12 N) at the beginning of both staircases and halved it during the first three reversals (6 N, 3 N, 1.5 N). After these three reductions, the step size was kept constant at 1.5 N. The experiment ended when there had been 13 inversions of answers (a positive response followed by a negative one or vice-versa) in both the ascending and the descending staircase. For each subject, the last 10 reversals of each staircase (depicted in bold in *Force Presentation* plot of Fig 2A for one subject) were averaged to extract individual ascending and descending PSE values. Since individual ascendant and descendent PSE values did not statistically differ (no parametric paired sample Wilcoxon Signed Rank test, n = 10, p < 0.05), they were averaged together to provide a unique PSE value for further analysis. Each recording session of muscles activity started after that participants remained within the tolerance target limits for more than 1 second.

### Data acquisition

Arm position was recorded using both an electro-magnetic motion tracking system (Mid-Range 3D Guidance Trackstar, Ascension, Footscray, Australia) and the Trackhold device (i.e., TH). With the former device, we tracked the position of the wrist (styloid process of the ulna), elbow (epicondylus lateralis), and shoulder (acromion) joints by means of three active markers with a sampling frequency of 80 Hz and a spatial resolution better than 2 mm. The TH device (100 Hz sampling rate) was used to track the point of force application *Xf* and we used this stream of data to update visual cursor position. Surface electromyography (EMG) was recorded by means of active bipolar surface electrodes (DE 2.1; Delsys, Boston, MA) from the following eight muscles: brachio-radialis (BrRad); biceps brachii, long head (Bic); triceps brachii, long head (Tric); deltoid, anterior (DeltA); deltoid, posterior (DeltP); trapezius middle (TrapM); trapezius upper (TrapU); latissimus dorsi (LatD). EMG signals were band-pass filtered (20–400 Hz) and amplified (total gain 1000, Bagnoli-16, Delsys Inc.). The signal was then digitized at 1 KHz (PCI-6035E, National Instruments, Austin, TX). EMG data acquisition and synchronization with motion tracking was performed with custom software written in LabView (National Instruments, Austin, TX). A custom XVR routine controlled the experiment by exchanging acknowledgments to a Matlab GUI (Mathworks, Natick, USA) that defined next trial stimulus to be presented; by sending triggers to LabView to start and stop EMG recording; by updating visual feedback position; and by logging the time of all relevant behavioral events (e.g., answer provided, target tolerance limits exceeded).

### Data analysis

#### Arm kinetic and EMG traces

All the analyses were performed with custom software written in Matlab (Mathworks, Natick, MA). Position data were low-pass filtered at 10 Hz with a second order, zero-phase shift Butterworth filter. The time varying position of the markers placed on the shoulder, elbow and wrist were used in post-processing analysis to estimate elbow and shoulder joint angles. We then used these measures to estimate the joint elbow and shoulder flexion torques for ten subjects. In particular a kinematic and kinetic model of the arm that incorporated geometrical and inertial parameters of arm segments was used to estimate, at each sample, the shoulder and elbow flexion angles as well as the joint total (net) torques actively generated by the subjects muscles around those joints (see [33] and paragraph 1.3 in S1 Text for details on the model and for its differences from our model). Residual torques were also calculated by subtracting the gravitational components from the net torque, which provide a measure of the joints moment changes exerted by the participant’s in response to the different external upward force applied.

The EMGs traces of each trial were digitally full-wave rectified and low pass filtered with a second order zero-phase shift Butterworth at 25 Hz. After careful visual inspections of both the position and EMG profiles, we chose not to consider for further analysis those trials where muscle waveforms showed artifacts or the cursor position exceeded tolerance target limits. On average across all subjects, the percentage of trials excluded was 2.4 ± 2.1% of all trials. Examples of the EMG signals we considered for the analysis are depicted in *EMG traces* plots of Fig 2B, that describes muscles activity of a typical subject when no external forces (i.e., 0 N) were applied on her arm. Individual Mean Absolute Values (MAV) of muscular activity were calculated for each EMG channel from all trials. Finally, MAV value of each muscle was normalized with respect to the maximum MAV recorded for that muscle across all presented forces (normMAV).

#### Muscle-metric curve extraction

The main goal of this study was to quantify how the activity of the recorded muscles could account for the concomitant psychophysical behavior during the force detection task. To this end, we had to produce a quantitative description of muscular activity (i.e., *muscle-metric*) that was directly comparable to the description of psychophysical decisions captured in the psychometric function. A description of the analytical steps carried out to obtain the muscle metric curve is provided in Fig 2B by plotting the data of one subject. For each trial, we first pooled together the normalized MAV of each EMG channel by considering their effective pulling direction in task coordinates (i.e., upward or downward directions) by means of the following equation:

$$PoolEMG_{i}=\sum_{i=1}^{n}\left(normMAV_{k}*w_{k}\right)$$ (1)

where *i* is the single trial, *k* is the single muscle channel, and *w* is the difference between the normMAV of muscle *i* when the upward force was highest (30 N) and the normMAV of the same muscle when the upward force was zero (0 N). Consequently, *w* is a coefficient whose sign corresponds to the direction of the muscle pulling action, *wk* < 0 characterizing an upward pulling, flexor, muscle and *wk* > 0 characterizing a downward pulling, extensor muscle. In addition, the absolute value of *w* is a measure of the sensitivity of muscle *k* with respect to the range of forces presented, where |*w|* = 0 indicates no modulation of the muscle *k* with respect to each upward forces presented. Therefore, high values of PoolEMG will be associated to a high joint extensor muscle activity (namely, high perceived value of the external upward force on arm and assumed psychophysical answers “YES”); while low PoolEMG will be related to a low joint extensor muscle action(namely, low upward force intensities and assumed psychophysical answers “NO”). Indeed, the first trials of the ascending staircase (low external forces, circles in *PoolEMG trend* plot of Fig 2B) produced low (even negative) PoolEMG, while the first trials of the descending staircase (high external forces, black squares in *PoolEMG trend* plot of Fig 2B) produced high values of PoolEMG. Therefore PoolEMG distribution (*PoolEMG distribution* plot of Fig 2B) can be interpreted as a continuum of the arm muscle activity associated with the external force presented; from the flexor (upward) muscular torque to support the arm with minimum external force level to the maximum extensor (downward) muscular torque with maximum external force (*PoolEMG UP and PoolEMG DOWN*, respectively in *PoolEMG distribution* plot of Fig 2B). Then, a muscle-metric function was computed to reflect the probability of observing a PoolEMG activity above a criterion level set to the mean of the PoolEMG distribution (*muscle-metric curve* plot of Fig 2B, see also paragraph 1.4 in S1 Text for details on criterion selection). We expected that this probability increased from zero to unity as the upward force level increased, and the resulting “muscle-metric curve” could be quantitatively compared to the psychometric function obtained from the probability of positive answer in the same task (upper, middle and lower curve plots in Figure B in S1 Text). We chose to model both the muscle and psycho metric functions by applying a Generalized Linear Model [34] to our data due to its advantageous feature of making no assumptions with regards to the shape of the response distribution (both PoolEMG and the force levels explored by the subjects). Our GLM model (Matlab Statistics Toolbox) had 1) the PooledEMG and the psychophysical YES/NO answer distributions as response variables, 2) the upward force intensity as linear predictor for both psychophysical decisions and PoolEMG values, 3) the logit as the link function relating the response variable to the linear predictor (*“GLM logistic fit”* in Fig 2A and 2B). Finally, we used the maximum likelihood estimation (MLE) method to extract the best fit parameters of each curve (*Psycho-metric curve* and *Muscle-metric curve* in Fig 2A and 2B). Slope (β1) and intercept (β0) values were used to compare the shapes of the two metric functions and to calculate the muscular (Ψ emg) and the psychophysical (Ψ ps) absolute threshold (i.e., PSE, Ψ = —β0/β1). Then, to quantify the concordance of the two metrics, we subtracted from the psychometric curve parameters and thresholds the same measures obtained from the muscle-metric curves (i.e., Δ intercept, slope and PSE). In order to compare the most reliable values obtained from the two data sets, we chose to compare curve parameters and PSE only for those subjects respecting two criteria of goodness of logit fit: *R*^*2*^ explaining psychophysical performance equal or higher than 0.6, and goodness of logit fit explaining perceptual and muscular probabilities merged together (*R² merged*), higher than 0.5. Instead, for those subjects whose curve describing perceptual performance had a fit lower than 0.6, we considered the PSE extracted by the up-down staircase method to compare the perceptual with the muscle-metric detection threshold.

#### Multiple regression of muscular activity

A further goal of the present study was to identify which arm muscles, among the eight considered, mostly contributed during the force detection task. To this end, the PooledEmg overall activity was submitted to a multiple regression analysis according to the model:

$$PooledEmg_{i}=\beta_{0}+\beta_{1}BrRad_{i}+\beta_{2}Bic_{i}+\beta_{3}Tric_{i}+\beta_{4}TrapM_{i}+\beta_{5}TrapU_{i}+\beta_{6}LatD_{i}+\beta_{7}DeltA_{i}+\beta_{8}DeltP_{i}$$ (2)

where PooledEmg at each trial *i* is predicted by the linear combination of the normMAV associated to each muscle. *βk* is the regression coefficient for each regression term (with 1 < *k* < 8) and *β0* is the regression constant term. Thus, for each subject we extracted eight *βk* standardized regression coefficients by standardizing all variable across trials before the regression, namely subtracting the mean and dividing by the standard deviation of each muscle. Positive *βk* represent direct relation between the increase of the upward external forces and the activity of the muscles with downward pulling direction (i.e., extensors). Conversely, negative coefficients indicate inverse relations between external forces and the activity of those muscles with upward pulling direction (i.e., flexors). Only *βk* that presented a statistically significant (*p* < 0.05) dependence on the stimulus force were considered.

### Simulations

Simulations on the empirical data were performed to investigate the reliability of the concordance between the two metric curves extracted as a function of the upward forces presented, and to explore whether different pooling of muscles might influence the level of explanation of the perceptual performance.

**Fig 3:**
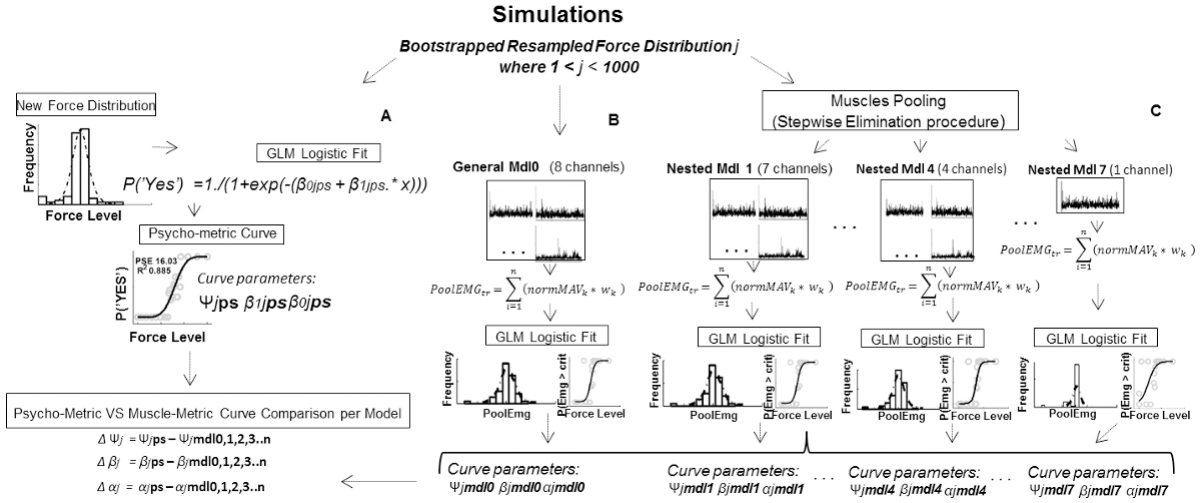
Bootstrap and Stepwise Elimination Procedures. **A)** For each of the 1000 simulated experiment (resampled stimulus force order) psychometric curve was obtained by fitting a logistic function to the probability of a positive answer with respect to each external force level (x). Ψ*ps*, *β1ps*, *β0ps* indicate the absolute threshold (PSE), the slope and the intercept, respectively, characterizing the logistic function that best explains the probability of detecting an upward force given each stimulus force. **B)** Computation of 1000 muscle-metric curves (one per each new force presentation order) obtained by means of a weighted pool of all 8 muscles activity (PoolEMG). **C)** Computation of 1000 muscle-metric curves (one per each new force presentation order) obtained from each nested model of the muscles activity pooled as above (PoolEMG), whose number of terms was iteratively reduced from 7 to 1. Once 1000 simulated experiments were performed per each nested model, muscle-metric and psycho-metric curves output were compared (bottom left).

The hierarchy of the simulation was defined by two sequential steps: the first aimed to define each one of the 8 muscular nested models (i.e., muscles pooling, Step-Wise Backward Elimination procedure), the second aimed to quantify the reliability of its associated muscle-metric parameters (i.e., bootstrap). As shown in Fig 3, each iteration *j* of the simulation was characterized by a parametric bootstrap resampling of the experimental force presentation order from which new (i.e., 1000) psychometric curve parameters and threshold were extracted (Fig 3A). The same simulated force presentation order was used to compute the muscle-metric curve associated to the PoolEMG distribution obtained from the pattern of eight muscles (i.e., *General Mdl0*, Fig 3B). After 1000 simulated experiments, each of them producing both a psycho- metric and muscle-metric curve associated with the eight muscles general model, a simplified nested regression model, was obtained by eliminating the least relevant -and/or not statistically significant- muscle term (lower *βn*, backward elimination, *Muscles Pooling* in Fig 3C). Then, new 1000 experiments were simulated and muscle-metric curves were extracted by using the PoolEMG distribution obtained by those muscles composing the simplified nested model. Thus, at each loop of such a two steps simulation procedure (bootstrap and backward elimination) a new simplified muscular pattern model M, with *i* minus 1 number of terms was provided—*i* being between 2 and 8 the number of terms of the preceding model-. Simulations stopped after 1000 muscle-metric curve of the nested model with only one regression term were extracted. In order to compare the reliability of the psycho vs muscle metrics concordance among muscular regression models the same sequence (i.e., seed) of random force presentation resampling was used for all models and it was changed per each subject (i.e., *rng* function, *twister* generator, Mathworks, Natick, MA).

This procedure allowed to obtain seven nested muscular models (i.e., muscle pooling, each one with the highest regression coefficients) extracted from the initial general model that considered all eight arm muscles recorded from the subject. We then compared, for each subject, the resulting seven nested models by taking into account their goodness of fit in explaining the individual PoolEMG total variance measured during the experiment and their value of the Bayesian Information Criterion (BIC [35], hereafter BaIC to differentiate it from bicep nomenclature, *Bic*). BaIC is a measure of both how much each model is less complex than the general one, and its likelihood to provide the observed empirical data (see paragraph 1.5 in S1 Text for details). To make the comparison among model BaIC values easier, the difference between the best BaIC obtained (i.e., BaIC ≈ 0) and each model BaIC (i.e., Delta BaIC) was normalized with respect to the highest Delta BaIC obtained among all models (Normalized Delta BaIC). Therefore, the best nested model was selected as that one that presented the normalized Delta BaIC values closest to zero, and an explanation of the empirical PoolEMG variance higher than 95%. Finally, we quantified the reliability of the concordance between each nested model muscle-metric curve and the psychometric curve by simulating 1000 experiments (i.e., boostrap parametric resampling of force presentation order) and extracting every time the explained variance (*R*^2^) of the muscle-metric curve in accounting for the probability of upward force detection.

## Results

### Perceptual performance

Individual psychophysical decisions with respect to upward force levels were described by fitting a logit function to the probability of positive answers (*Psycho-metric curve* plot in Fig 2A). The curve explained relatively well the individual probabilities of reporting a detection of upward force (answer “YES”) for each force presented. Specifically, we observed an average, across subjects, goodness of fit (*R*^*2*^) value and standard deviation of 0.77 ± 0.16. In the first three rows of Table 1 we reported the maximum likelihood (ML) estimated absolute thresholds (i.e., PSE), intercepts and slopes (± SE) calculated for each subject and characterizing the psychometric curves. As shown in the fourth row of the table (*R*^*2*^ *psy*), 3 subjects out of 14 showed a goodness of fit lower than 0.6 (mean *R*^*2*^ *psy* and SD of 0.55 ± 0.05). For these subjects, we did not consider their psychometric curve parameters but only their PSE extracted by the up-down staircase method (i.e., *PSE stair* in Table 1). In accordance with previous works that showed no statistically significant differences between perceptual thresholds obtained by the up-down staircase method and by ML estimation [36], we found that our PSEs extracted by logit fitting (subjects with *R*^*2*^ *psy* ≥ 0.6) and from averaging reversals did not differ in a statistically significant manner (Wilcoxon Signed non parametric Test, *n = 11*, *signedrank* = 37.5 with *p* = 0.72). Such an outcome allowed us to use, in further analysis, the PSE stair obtained from staircase method when *R*^*2*^ *psy* was not reliable (*R*^*2*^ < 0.6). Individual parameter values that we considered for further analysis are reported in bold in Table 1.

**Table 1:** Psycho-Metric Curve Parameters. Individual maximum likelihood (ML) estimated threshold (*PSE fit*), intercept and slope ± SE obtained by Logit glm fit describing the percentage of positive answers as function of upward force level. *R*^*2*^ *psy* values describe the explained variance of the Logit function used to describe psychophysical decisions per each subject. *PSE stair* values were obtained by averaging the force levels associated with the last 10 reversals of the up-down staircase method. Bold values are those considered for further analysis. Either *PSE fit* or *PSE stairs* were used whether individual *R*^*2*^ *psy* were higher (as well as equal) or lower than 0.60, respectively.

	*Subj*.*1*	*Subj*.*2*	*Subj*.*3*	*Subj*.*4*	*Subj*.*5*	*Subj*.*6*	*Subj*.*7*	*Subj*.*8*	*Subj*.*9*	*Subj*.*10*	*Subj*.*11*	*Subj*.*12*	*Subj*.*13*	*Subj*.*14*
*PSE fit (N)*	**16.0±0.5**	11.4±1	**9.6±0.2**	**2.3±0.5**	0.8±0.9	**8.3±0.5**	1.0±0.5	**0.1±0.2**	**6.0±0.3**	**8.9±0.2**	**11.0±0.4**	**11.4±0.9**	**18.8±0.5**	**3.9±0.1**
*Intercept*	**-6.7±2.2**	-2.2±0,7	**-10.5±3.7**	**-1.1±0.6**	0.0±0.3	**-2.9±1.3**	-0.4±0.3	**-0.2±0.3**	**-4.5±1.5**	**-12.1±3.7**	**-6.6±2.4**	**-2.3±0.8**	**-6.2±2.1**	**-7.5±2.3**
*Slope*	**0.4±0.1**	0.2±0.1	**1.1±0.4**	**0.4±0.2**	0.19±0.1	**0.3±0.1**	0.44±0.3	**1.3±0.5**	**0.7±0.2**	**1.3±0.4**	**0.6±0.2**	**0.2±0.1**	**0.3±0.1**	**1.9±0.6**
*R² psy*	0.88	0.59	0.93	0.66	0.49	0.78	0.56	0.77	0.95	0.98	0.85	0.66	0.82	0.96
*PSE Stair (N)*	15.3±0.6	**8.9±1.0**	10±0.3	2.2±0.4	**1.9±0.4**	8.4±0.3	**0.9±0.4**	0.1±0.3	5.6±0.3	8.9±0.2	11.6±0.6	10.9±0.7	19±0.6	4.0±0.3

### Muscular performance

The method that we used to quantify the pooled arm muscular activity with respect to each upward force level allowed us to compare the trend of psychophysical decisions with the trend of arm muscular pulls across trials (compare *Force Presentation* plot with *PoolEMG trend* plot of one subject in Fig 2A and 2B). Indeed, we observed a statistically significant correlation for 12 subjects (p < 0.05) out of 14 between the changes of the forces explored by the subjects under the up-down procedure and the changes of the PoolEMG at each trial (mean Pearson *rho* 0.73 with SD 0.18). Similarly the muscle-metric curve computed by fitting a logistic function to the probability of PoolEmg above the criterion level provided an overall good fit across subjects. In fact, with the exception of participants 9 and 6 for whom logit function had no explanations of the PoolEMG modulation (*R*^*2*^ *emg* ≈ 0 in Table 2), we obtained an average goodness of logit fit (*R*^*2*^ *emg*, fourth row in Table 2) of 0.72 with a SD of 0.16. This observation ensured us that the choice of logistic as link function provided a satisfactory description of the global muscular activity with respect to force level. Bold values in the first three rows of Table 2 are the individual ML estimated muscle-metric curve parameters and absolute thresholds considered for further analysis. Participants 6, 9 and 7 were excluded from successive comparisons because of their less reliable fitting (see *Muscle-Metric Curve Extraction* in Material and Methods).

**Table 2:** Muscular-Metric Parameters. Individual ML threshold, intercept and slope parameters ± SE obtained by Logit glm fit describing the percentage of muscular activation (namely, PoolEmg > criterion) as function of upward force level presentation. R^2^ emg values describe, per each subject, the explained variance of the Logit function used to account for the probability of PoolEmg > criterion at each force presented. An index of similarity (*R*^*2*^ *merged*) between the two metrics was defined as the goodness of fit of a Logit function explaining both perceptual and muscular probabilities merged together. Thus, R^2^ merged represents the level of concordance between muscular and perceptual performances in describing the subjects’ decision about upward forces. Participants 6 and 9 were excluded from further analysis since Logit function could not reliably explained their probability of pooled muscular activation over criterion (R^2^ ≈ 0). Similarly, subject 7 was excluded since his index of concordance between muscular activity and perceptual decisions was lower than 0.5.

	*Subj*.*1*	*Subj*.*2*	*Subj*.*3*	*Subj*.*4*	*Subj*.*5*	*Subj*.*6*	*Subj*.*7*	*Subj*.*8*	*Subj*.*9*	*Subj*.*10*	*Subj*.*11*	*Subj*.*12*	*Subj*.*13*	*Subj*.*14*
*PSE (N)*	**14.5±0.2**	**8.6±0.2**	**8.9±0.2**	**6.6±1.0**	**4.2±0.2**	48±10	0.1±1.2	**1.2±0.2**	-14±15	**8.5±0.5**	**11.9±0.3**	**11.3±0.5**	**17.1±0.3**	**3.3±0.6**
*Intercept*	**-14.4±3.8**	**-7.7±1.7**	**-12±3.9**	**-1.4±0.4**	**-3.3±0.7**	-1±0.5	-0.0±0.3	**-1.1±0.4**	-0.2±0.4	**-3.8±2**	**-9.2±2.6**	**-3.9±1.4**	**-11.7±3.7**	**-1.4±1.0**
*Slope*	**1±0.2**	**0.9±1.9**	**1.3±0.4**	**0.2±0.1**	**0.8±0.2**	0.0±0.0	0.2±0.1	**0.9±0.3**	0.0±0,0	**0.4±0.2**	**0.7±0.2**	**0.3±0.1**	**0.7±0.2**	**0.4±0.2**
*R² emg*	0.77	0.81	0.92	0.64	0.87	-0.04	0.45	0.75	0.02	0.7	0.8	0.4	0.89	0.7
*R² merged*	0.75	0.63	0.92	0.6	0.65	0.15	0.49	0.69	0.27	0.83	0.83	0.56	0.83	0.79

### Psycho-metric versus muscle-metric performance

**Fig 4:**
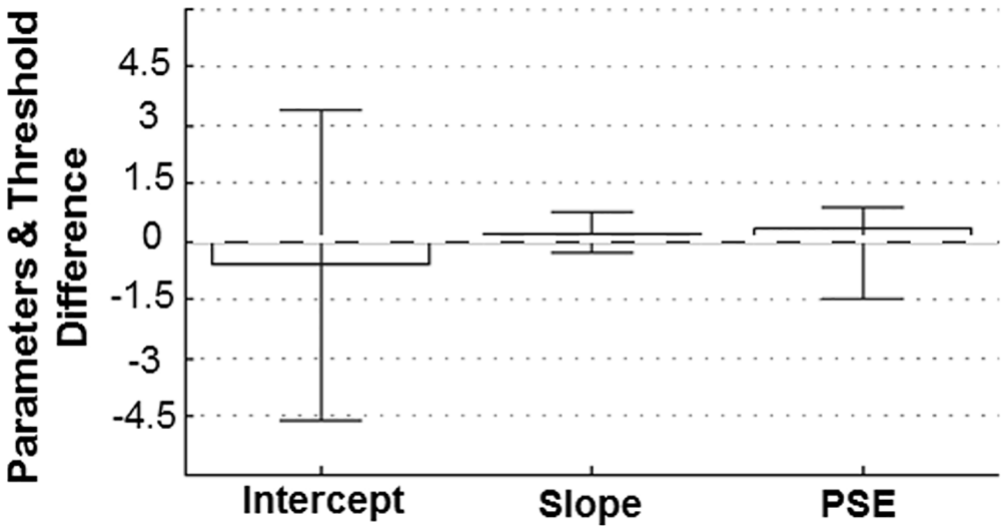
Psycho-metric vs Muscle-metric Parameters Similarity. Concordance between the shape of the psycho and muscle metric curves as well as between thresholds. Each bar shows the across subjects averaged difference values (perceptual—muscular) between the psychometric and muscle-metric datasets with 95% confidence intervals (CI). Zero difference values (horizontal dashed line) indicates a perfect match between psychometric and muscle-metric curves as well as relative thresholds. PSE difference below zero indicates that muscles tended to have higher thresholds than perceptual ones. Curve parameters difference mean value and CIs were averaged across 9 subjects (those with *R*^*2*^ *merged >* 0.5 and *R*^*2*^ *psy ≥* 0.6, Tables 1 and 2). PSE differences were averaged across 11 subjects (*R*^*2*^ *merged >* 0.5, values in bold Table 2) whose thresholds and CIs were calculated by considering PSE values from reversals when *R*^*2*^ *psy* was lower than 0.6.

A Two Sample Kolmogorov-Smirnov test was submitted to each subject’s distribution of the probability of PoolEMG above the criterion and the probability of positive answers. We found that, for 11 participants out of 14, both sets of probabilities could be described by the same distribution (Kolmogorov-Smirnov, *p > 0*.*05*, for 11 subjects and *p = 0*.*00*, *p = 0*.*01*, *p = 0*.*02* for subjects 9, 6 and 5, respectively). Furthermore, we assessed the similarity of the psychometric and muscle-metric curves by fitting a logit function to the two probabilities data set pooled together and by looking at its level of explained variance (*R*^*2*^ *merged*, last row of Table 2). As shown by *R*^*2*^ *merged* values reported in the Table, the explained variance of a single function accounting for the two (psychophysical and muscle-metrical) data sets did not decrease significantly with respect to the *R*^*2*^ *psy* values (compare individual *R*^*2*^ *merged* values reported in Table 2 with *R*^*2*^ *psy* reported in Table 1). No statistical differences were found by performing a Wilcoxon Signed non parametric test on the two groups of goodness of fits of those subjects with *R*^*2*^ *merged* higher than 0.5 (n = 11, *signedrank* = 50.5 with *p* = 0.13). Such a statistical result suggests that, for 78% of our whole sample, the trial-by-trial muscular performance is statistically indistinguishable from the psychophysical performance. A quantification of the predictive power of the muscle-metric curve about the psychophysical decisions is depicted in Fig 4. Bars represent the average difference values (psychometric minus muscle-metric estimates) of the intercepts, slopes and thresholds across subjects with their 95% confidence intervals (CI). Interestingly, the CIs of the differences of both curve parameters and thresholds included the zero value, indicating a very good match between the two curves. Specifically, the differences in the curve parameters were -0.60 (lower CI: -4.6; upper CI: 3.4) and 0.18 (-0.34; 0.70) for intercept and slope, respectively. The differences between perceptual and muscular detection thresholds were measured by considering *PSE stair* values, instead of *PSE fit*, for those participants having a *R*^*2*^ *psy* lower than 0.6. As shown in Fig 4, averaged PSE difference was 0.31 (-1.48; 0.88). The higher distance from zero of the lower confidence interval indicates that muscles tended to be less sensitive than perceptual judgments, the former being characterized by a higher PSE. The observed concordance between muscular and perceptual behavior shows that perceptual detection of unloading forces are strongly related to the accompanying patterns of muscle activation. This observation in turn suggests that unloading force detection is driven by an efferent copy of the descending motor commands generating the change in muscular activity associated with each stimulus force.

### Extensors and flexors *W* coefficients

**Fig 5:**
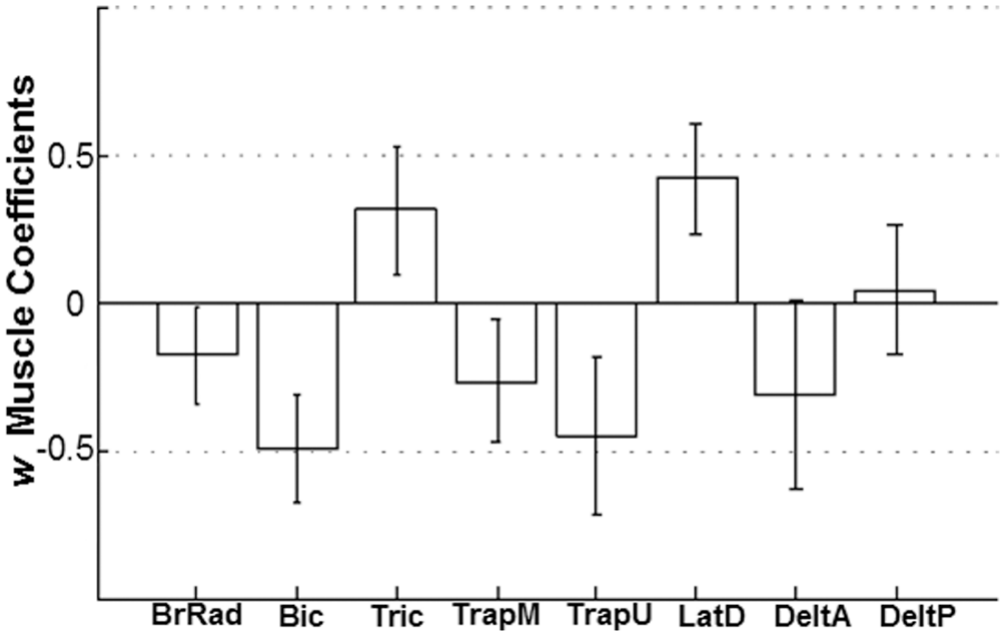
*W* coefficients per each muscle. Mean *w* coefficients for each muscle ± SD across subjects. Coefficient values represent an estimate of the sensitivity of each muscle to be modulated by the changes in the external forces. Positive values correspond to joint extensor modulations in response to changes in the upward external force. Negative coefficients quantified the correspondence between joint flexor muscles and external force changes.

With the aim of analyzing the sensitivity of each muscle to be modulated with respect to the range of forces presented, we looked at the associated *w* coefficients calculated as reported in ‘Muscle Metric Curve Extraction’ (Data Analysis section). Mean ± SD coefficients values calculated across subjects, reported in Fig 5, show clearly that flexor muscles (negative coefficients) presented a higher sensitivity to the upward force modulation than the extensor muscles (positive coefficients). In particular Bic and TrapU provided the highest mean ± SD *w* coefficient values, -0.5 ± 0.18 and -0.45 ± 0.26, respectively. The fact that the most relevant flexor muscles were associated with both shoulder and elbow joints suggests a similar contribution of muscle torques at both joints in the unloading force detection task. On the other hand, elbow and shoulder extensor muscles appeared to be less relevant (0.31 ± 0.22 and 0.42 ± 0.18 for Tric, long head, and LatD, respectively), but once again with a contribution at both shoulder and elbow joints. DeltP was the unique muscle showing an ambiguous modulation, across subjects, with both joint flexor and extensor activity (SD centered in zero). For some participants the external force changes had no effect on BrRad and DeltA activity. Overall, participants appeared to modulate to a similar extent both elbow and shoulder muscle torque in response to the external upward force, with a greater extent of modulation of flexor muscles than extensor muscles.

### Inter-individual differences

**Fig 6:**
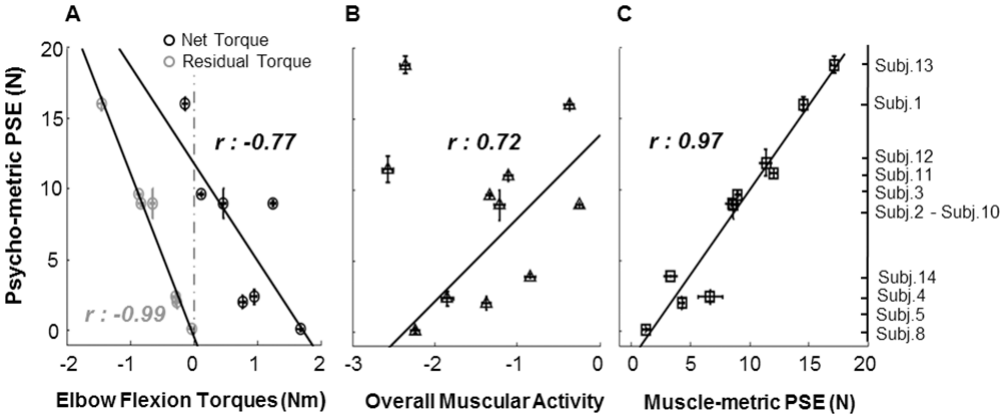
Individual psychometric thresholds as function of individual elbow joint torques, overall muscular activity and muscle-metric PSE. **A)** Individual median (± S.E.) of net (black) and residual (gray) elbow flexion torques as function of individual psychophysical PSE (± S.E.) of those subjects that respected goodness of fit criteria and joint torques were extracted. Residual torque values were obtained by removing the gravitational component from the total net torque. Negative torques indicate downward resultant joint torque. Positive values represent resultant upward joint torques. Continuous lines and *r* values are the best fitting regression line and the resultant Pearson correlation through the data points, respectively. **B)** PSE values as function of individual overall muscular activity calculated by extracting the median of the sum of the normalized MAV value among muscles at each trial for all subjects showing *R² merged >* 0.5. Negative values indicate main flexor overall muscular activity, while positive represent overall extensor activity. Pearson correlation and best fitting regression line were extracted excluding subject 12 and 13. **C)** Correlation among individual psychophysical PSE (± S.E.) and PSE (± S.E.) extracted from muscle-metric curve (same data as in Fig 4). In all plots, *r* values represent statistically significant Pearson correlation (*p*< 0.05).

As it can be noticed from Table 1, the psychophysical *PSE* values we considered (values reported in bold in the first and last rows of the table) cover a wide range of values across subjects, from 0.1 N to 18.8 N. In practice, subjects perceived the lowest detectable upward force on their arm (i.e., PSE) at widely different stimulus intensities. This finding suggests that participants may have used different strategies to judge the external forces and make a perceptual decision. The three plots depicted in Fig 6 describe the outcome of our analysis aimed to investigate the variables that mainly correlated with the inter-individual differences in psychophysical sensitivity. In general, we found a highly significant correlation between the inter-subjects variability in the PSEs and the variability of the total (net) and residual elbow flexion torques, *r*: -0.77 (n = 7, *p* = 0.04) and *r*: -0.99 (n = 7, p = 0.00), respectively. Correlation values measured between individual PSEs and participants’ shoulder flexion residual torques (not illustrated) were similar to those observed with elbow torques (n = 7, *r*: -0.98, *p* = 0.00), however the differences among subjects’ PSEs poorly correlated with their net shoulder torques (n = 7, *r*: -0.43, *p* = 0.37) and resulted not statistically significant.

#### Flexion joint torques vs psycho-metric PSEs

As shown in Fig 6A, the reduction of elbow joint residual torque (median ± SE), as well as for the not illustrated shoulder torque, highly correlates with the decrease of the individual PSE. In particular, among the participants for whom joint torques were computed, those who showed the lowest PSE values were also those who presented the flexion residual torques closest to zero (gray circles, subjects 8, 5 and 4, Fig 6A). In other words, it seems that these subjects judged the intensity of the external forces without taking into account the muscular gravitational torque necessary to sustain the arm against gravity but interpreting the lowest upward external force as that one implying the lowest, null, residual elbow flexion torque. Interestingly, other participants reported the upward external forces to be at minimum level (i.e., PSE) although the actual upward force applied on the arm did not imply a null contribution of the residual torque to the total elbow exerted moment (gray circles subjects 1 and 3, Fig 6A). Indeed Fig 6A shows that the subjects with high PSE values presented the net total torques closest to zero (black circles, subjects 1and 3). Therefore these subjects appear to take into account also the gravitational torque acting on their arm to detect the minimum intensity of the external upward force. Indeed these measures lead to hypothesize that the strategy of this latter group of participants was to judge the upward external force in terms of its facilitation to compensate the effects of gravity acting on arm (i.e., lowest upward force ≈ lowest net torque). Interestingly, Fig 6A also shows that apart from these two distinct perceptual behaviors, some other subjects reported the upward external forces to be at lowest level even if neither total nor residual torques were approaching zero (e.g., subject 10). For these subjects, an intermediate strategy, where both gravitational and total torques are taken into account, seems to have been used. We will further address this observation in the Discussion section.

#### Overall muscular activity vs psycho-metric PSEs

The direct relation observed in most of the subjects (having *R*^*2*^ *merged > 0*.*5*) between the individual overall muscular activity and the psychophysical PSEs gives further supports to the hypothesis of different participants’ strategies. The individual median ± S.E. of the overall muscular activity shown in Fig 6B was extracted by summing all muscles normMAV together per each trial, where the sign of each channel normMAV was defined by the sign of the coefficient ‘*w*’ used in Eq 1. Since it does not consider the absolute values of *w*, this measure describes the global arm muscular activity regardless of the different amount of relation between each muscle and the external forces. Thus, as above negative and positive values represent upward (flexor) and downward (extensor) muscles activity, respectively. In Fig 6B it can be noticed that the PSE values of 9 out of 11 subjects increase as the overall muscular activity approaches zero (n = 9, *r*: 0.72, p = 0.03). In fact, those subjects showing the highest PSEs are those presenting the overall activity closest to zero, while those showing the lowest PSEs values have highest negative -flexor—activity. Moreover the across subjects overall decrease of muscles flexion activity—i.e., overall muscular activity approaching zero- is in agreement with the correlation depicted in Fig 6A between the flexion torques and psychophysical PSEs. In fact, the more participants exploited the external upward force to facilitate their muscular gravitational torques (i.e., higher PSE), the less the overall muscular activity was characterized by flexors muscles (i.e., less negative values, low net torque). Conversely, the amount of muscular flexion torque increased as much as subjects did not consider the modulation of the gravitational component as a variable to judge the external upward force (i.e., lower PSE, higher negative overall muscular values and low residual torque). Nevertheless, it ought to be noted that 2 participants’ overall muscular activity—i.e., subject 12 and 13- did not follow the same trend observed in all other subjects. A possible explanation of this finding might be that those subjects mostly relied on other extensor muscles different from those recorded in this study (e.g., Pectoralis). In fact, in these cases the overall muscular activity remained high despite of the high PSE probably because the summed amount of extensor activity (positive values) would poorly influence the global muscular activation that was mainly described by the flexors (negative). As a whole, the relation between the measures of the global muscular activity and the PSE variability lead to hypothesize that the different strategies used by participants mostly relied on the different interpretation of the combination between the external upward force and the muscular gravitational torque needed to complete the task.

#### Muscle-metric PSEs vs psycho-metric PSEs

Fig 6C depicts individual psychometric thresholds as function of the thresholds extracted by the muscle-metric curves. The figure clearly shows that the inter-subjects differences (± S.E.) in psychophysical sensitivity are fairly well predicted by muscle sensitivity (± S.E.). Indeed, the direct relation between the two data sets (same subjects as in Fig 6B) produced a statistically significant Pearson correlation (n = 11, *r*: 0.97, p = 0.00). Interestingly the concordance between psycho and muscle metric PSEs was also found for those subjects whose overall muscular activity did not follow other subjects’ trend (subject 12 and 13 in Fig 6B). This latter observation leads to claim that the steps taken to compute the muscle-metric curve, such as by taking into account the relation of each muscle with the external forces (i.e., |*w|* coefficient) and by considering the whole distribution of the poolEMG activity (i.e., p(PoolEMG > criterion)), might compensate for the consideration of those muscles that were no relevant to the perceptual task. As a whole the consistent ordinal relationship between muscular and psychophysical thresholds for 78% of our sample supports the hypothesis that force detection required in our task were mostly driven by the corticospinal motor commands sent to produce specific joint-muscular torques to both compensate gravity and counteract the external force.

### Simulations

#### Best model of muscular pattern

The statistics performed on each muscular nested model (data across subjects) aimed to identify the muscular pattern that best accounted for the PoolEMG variance (R^2^ PoolEMG) and presented a BaIC value closest to zero (see Figure C in S1 Text). As expected, the average across subjects normalized delta BaIC values showed a reduction as a function of the increased simplicity of each nested model. Indeed the normalized delta BaIC closest to zero involved three, two and one muscles as predictors (norm delta BaIC values = 0.08, 0.04, 0.0 respectively). In particular, only the model composed by three muscles (see paragraph 1.7 in S1 Text for the muscles with highest probabilities to compose this model) provided an explanation of the total variance of the PoolEMG higher than 95% (median across subjects R^2^ PoolEMG = 0.97 ± 0.03, *Lower graph* Figure C in S1 Text), whereas the nested models with two and one predictor presented a R^2^ PoolEMG of 0.92 ± 0.04 and 0.85 ± 0.09, respectively. Therefore, we selected the three term nested model (i.e., BEST) as the one providing a lower level of complexity than the general model with eight predictors and, at the same time, accounting for more than the 95% of the global arm muscular activity variance.

#### Nested muscular models accounting for psychophysical performance

**Fig 7:**
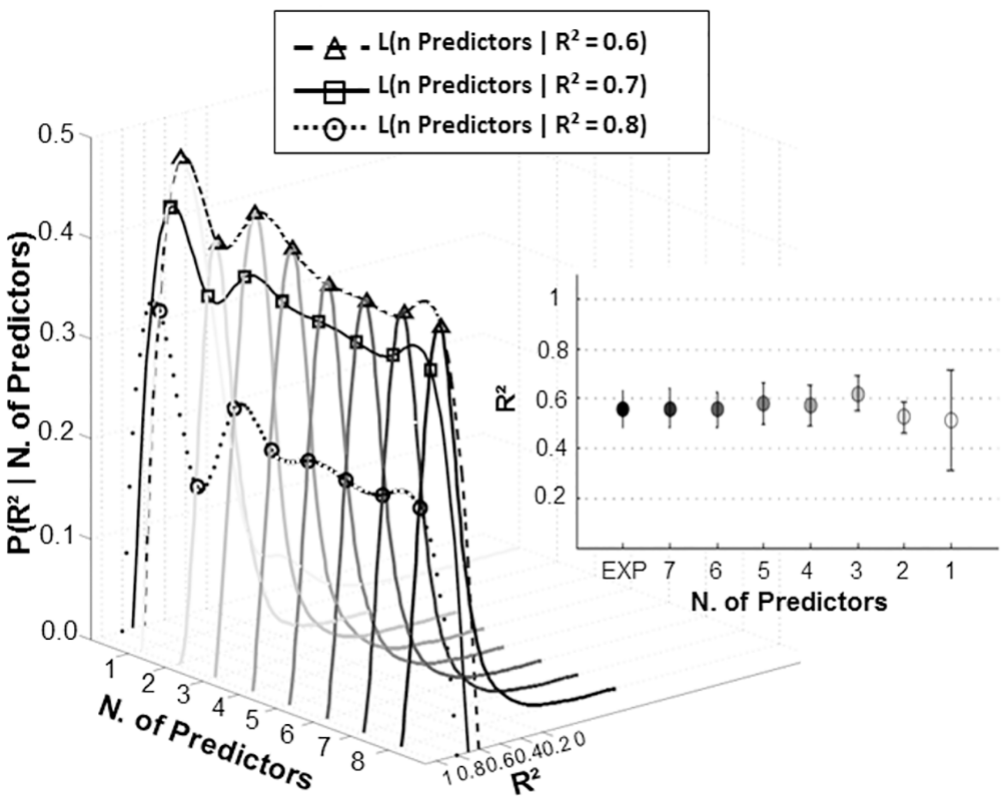
Probability density function of *R*^*2*^ values per each nested model. Each curve illustrates the distribution of R^2^ measures given each nested model’s number of predictors. R^2^ values quantify to what extent the muscle-metric curve, obtained from each nested model output, is able to account for psychophysical decisions. Triangles, squares and circles as well as dashed, continuous and dotted lines indicate the probability (i.e., Likelihood) of each model to provide predictions about the psychophysical decision with a coefficient of determination of 0.6, 0.7 and 0.8, respectively. *R²* distributions were obtained by merging together the simulation outcomes of the same 9 subjects considered in Fig 4. *Inset graph* depicts across subjects median and SE of R^2^ values from bootstrap resampling, obtained by calculating the explained variance of the muscle-metric curve in accounting for the probability of upward force detection. The model with 8 predictors (i.e., *EXP*) represents the empirical data.

The main plot and the inset graph of Fig 7 depict the whole distributions and the median ± SE of the *R*^2^ values quantifying the explained variance of the muscle-metric curve in accounting for the probability of upward force detection (same subjects as in Fig 4 merged together). Although the *R*^2^ associated to our best three terms muscular model was not statistically different from all other values (no parametric Kruskal–Wallis Anova Test, χ^2^ = 2, dof = 64, *p* = 0.96), it presented the higher coefficient of determination (3 n. of predictors in Fig 7). Conversely, the lowest and more variable *R*^*2*^ value was obtained with the output of the simplest, least, nested model (1 n. of predictors in Fig 7). Specifically, the explained variance of the perceptual performance accounted by all models ranged from 0.51 ± 0.20 to 0.62 ± 0.07, having the 1 term and 3 terms nested model the lowest and the highest predictive power, respectively. From the curves presented in Fig 7 we also extracted the likelihood of each model to provide a specific coefficient of determination (triangles, squares and circles in Fig 7). Likelihood values provide a clear idea of which model will more likely account for psychophysical performance with a coefficient of determination of 0.6, 0.7, 0.8. To test any differences among the likelihood of providing a specific *R*^*2*^, a Kruskal-Wallis non-parametric test with Tukey’s post-hoc comparisons was submitted on all models likelihoods, grouped for the three *R*^*2*^ target. No statistical differences between the likelihood of providing an *R*^*2*^ of 0.6 and 0.7 (*p* > 0.5) was found. Conversely the models likelihood of a *R*^*2*^ equal to 0.8 was significantly lower than that of providing the two previous coefficient of determinations (see also Figure D and paragraph 1.6 in S1 Text).

#### Reliability of muscle vs psycho–metric curves concordance

**Fig 8:**
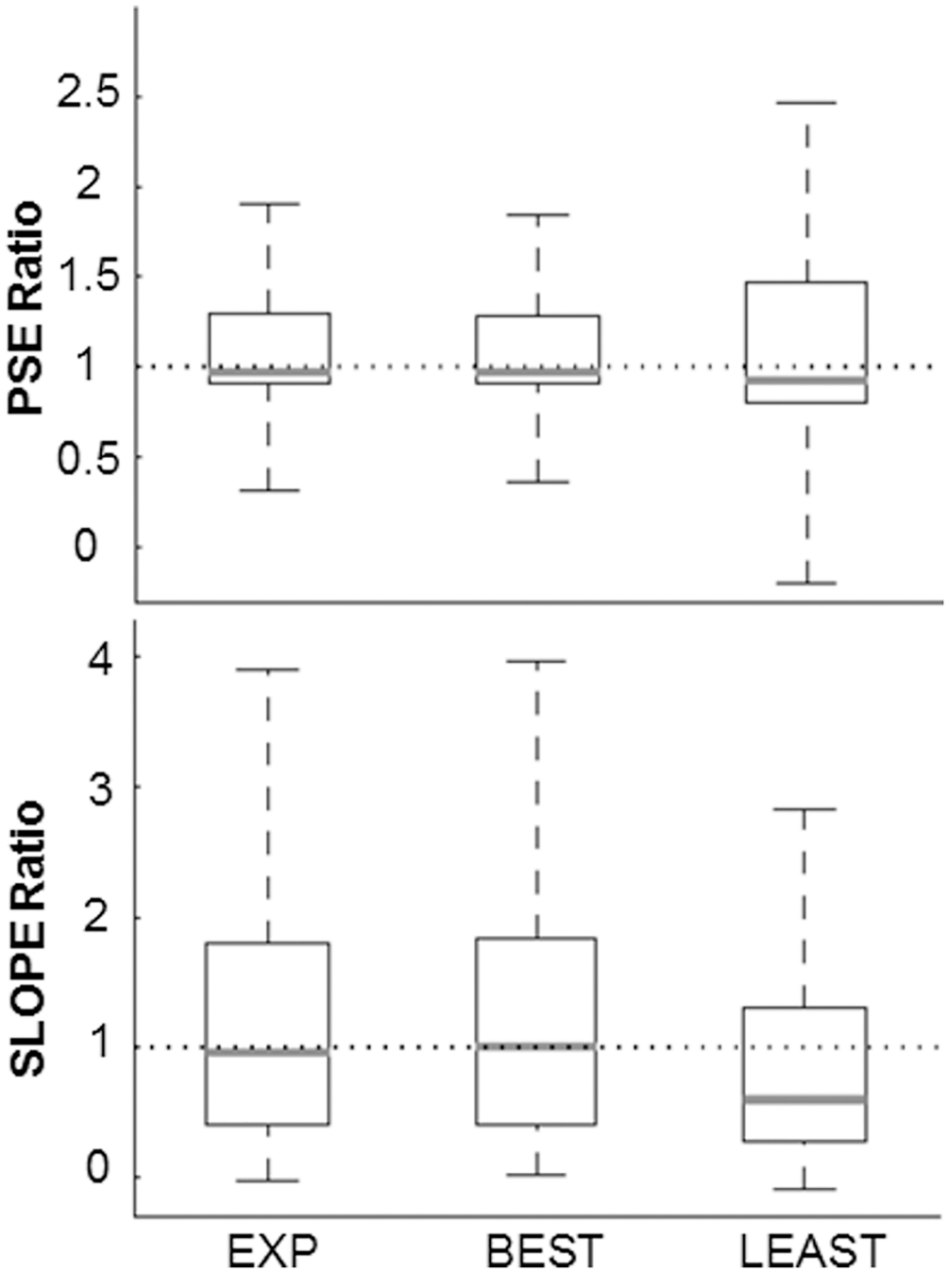
Threshold and slope ratios (muscle/behavior) of experimental data and two simulated models. *Upper graph* shows the ratio between the psychophysical threshold and the muscle-metric PSE from the empirical muscular model with 8 predictors (EXP), the simplest and reliable model selected with 3 predictors (BEST) and the model with just one predictors (LEAST). Data samples were obtained by merging 11 subjects (same as in Fig 4) PSEs from muscle and psycho -metric measures calculated across bootstrap procedure. *Lower graph* depicts the ratio between the slopes of the two metrics curves for the three muscular models mentioned above. Data samples were obtained by merging 9 subjects (same as in Fig 4) slopes muscle and psycho -metric measures calculated across bootstrap procedure. In each graphs gray lines indicate the medians of the ratio sample (50° percentile). Boxes represent data between the 25°, Q1, and 75°, Q3, percentile of the whole sample. Upper and lower whiskers size were calculated considering the values extracted from Q3 + 1.5 * (Q3-Q1) and Q3–1.5 * (Q3-Q1), respectively.

In agreement with the neuro-metric literature, we quantified the similarity between the muscle-metric and the psycho-metric functions by calculating a ratio for both the absolute thresholds and the shape of the curves (i.e., slope) obtained by the two metrics, where the greater is the concordance between the two curves, the closer the ratio will be to unity. Fig 8 depicts PSE and slope ratios obtained by merging the thresholds and the slopes of 11 and 9 subjects (same subjects as in Fig 4), respectively. In both graphs, the three boxplots represent the absolute thresholds and the shape of the curves obtained by bootstrapping the psychometric and muscle-metric data from the real experiment (*EXP*, eight terms muscular model), the best nested muscular model (*BEST*, three terms nested model) and the least muscular model composed by only one muscle (*LEAST*). As it can be noticed in the upper graph, the PSEs extracted from both the experimental and the best nested muscular model show a similar good concordance with the psychophysical thresholds in terms of PSE ratio median (0.972 and 0.975 for EXP and BEST, respectively), inter-quantiles difference (0.39 and 0.38) as well as upper (1.89 and 1.86) and lower (0.31 and 0.35) bounds. Instead the LEAST muscular model provided a slight reduction in the PSE ratio median (0.92) and a relevant increase in both the inter-quantiles difference (0.67) and the upper lower bounds (2.48 and -0.2, respectively). Overall, the PSE ratios suggest that the muscle metric curves obtained from the experimental and the best nested model provided a reliable concordance with the psychometric curve. Moreover in the boxplots of all PSE ratios the higher distance of the median from the third quartile indicates a tendency of the muscles to provide an absolute thresholds higher than the psychophysical ones (i.e., positive ratio). Likewise the PSE ratio, EXP and BEST models yielded a very similar slope ratio distribution. Again, in this case the median of the ratio approximated unity (0.97 and 1 for EXP and BEST, respectively), but it presented an increase of the inter-quantile difference (1.41 and 1.43) and the upper (3.93 and 4.00) and lower bounds (-0.04 and 0.01). On the contrary, LEAST nested model presented lower dispersion in the distribution of its slope ratios but with a relevant reduction in the median (0.59). In sum, the differences found in the slope ratio distributions indicate again a better muscle-perception concordance between the muscle metric curves obtained with either all eight or the three, most relevant, muscles. However the high dispersion of the data indicates a relevant difference between the variability of the muscles versus the psycho-metric curves. In particular the consistent presence of above unity slope ratios suggests higher variability (i.e., lower precision) in the perceptual decisions rather than in muscular estimates.

## Discussion

Participants were asked to detect the presence of an upward force applied on their arm while the EMG activity of 8 upper limb muscles was simultaneously recorded. By modeling the EMG changes with respect to the forces applied on their arm, we were able, for first time, to provide a quantitative description of the muscular activity (“*muscle-metric curve*”) that was directly comparable to the description of the psychophysical judgments described by the psychometric function. At least to the best of our knowledge, a representation of the mean EMG activity that captures their trial-by-trial modulation associated with the trial-by-trial psychophysical judgments has not been published yet. In their pioneering study, Sanes and Shadmher [11,12] examined the relationship between the changes in the activity of wrist flexor/extensor muscles and the associated psychophysical decisions during a uni-manual postural maintenance task. These authors showed the predictive capability of the muscles by identifying the proportions of trials in which a perceived increase of the stimulus force was accompanied by a change in EMG activity that exceeded a given threshold (i.e., half of the average difference of the activity produced during two consecutive constant flexor loads). The authors argued in favor of the idea of alternate, efferent/afferent signals dependent mechanisms for the sense of muscular effort by reporting both similarity and differences in the concordance/discordance index measured on de-afferented patients and controls. However, they did not provide any metrics to compare muscular with psychophysical performances in terms of precision and accuracy.

Here we showed that the changes of the pattern of muscular activity described by the muscle-metric curves could predict relatively well both the shape (precision) and the absolute position (accuracy) of the psychometric functions (Figs 4 and 8). Indeed, for 78% of our whole sample of subjects, a single function fitting both muscular and psychophysical performance was not statistically different from the function describing the psychophysical behavior alone. Our findings indicate, on the one hand, a strong correspondence between muscular activity and the final perceptual decision about force. Indeed the fact that muscle-metric curves were able to explain the wide differences observed in the individual perceptual thresholds suggests that the motor output drives the perception of force, perhaps to a greater extent than does the sensory input. On the other hand, by showing that a measure of motor output, such as electromyography, is able to describe fairly well psychophysical performances provides insights into the neural processes underpinning force perception. In the following paragraphs we separately address these two issues.

### Central contribution to force detection or biomechanical epiphenomenon?

In the present study the required psychophysical task entailed a direct relation between the forces applied and subject’s arm biomechanical behavior. In fact, subjects, to maintain arm posture, were constrained to modulate their muscular activity in accordance to the different upward forces applied. Since the aim of the study was to investigate the muscle-perception comparisons, in accordance with neuro-metric approach, it was crucial that the performance of the muscles was considered under the same constraints as the perceptual behavior [25]. Here we gave evidence that the processes of perceptual decisions about a force applied on arm might be partially explained by the CNS interpretation of the descending motor command involved in interacting with that force.

However, our findings might be also reasonably interpreted as an epiphenomenon emerging from the strong relation between the forces applied on arm and the biomechanical behavior they elicited to maintain the posture. If this pure biomechanical explanation hypothesis of our findings were correct, our results would show: 1) that participants interacting with similar PSE forces were constrained by the protocol to modulate their muscular activity in a similar fashion and 2) that since *p*(PoolEMG > criterion) is assumed to be driven by the distribution of the applied forces, that is modulated with respect to subject’s answers (adaptive staircase method), the resultant muscle-metric curve has to be highly predictive about the probabilities of the perceptual decision *p*(‘YES’).

**Fig 9:**
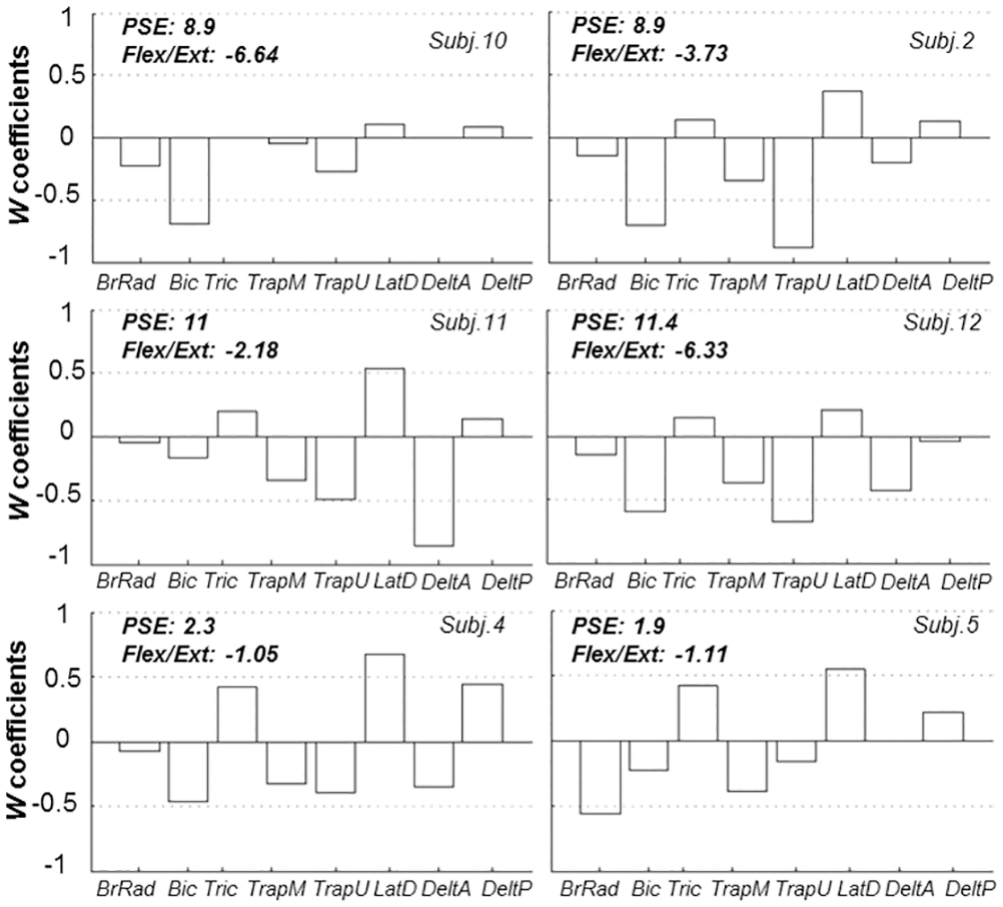
Individual distribution of muscular *w* coefficients and flexor/extensor ratio for pair of subjects with similar PSE. Each bar represents the w coefficient associated for each muscle. Coefficient value represent an estimate of the sensitivity of each muscle to be modulated by the changes in the external forces. Positive values correspond to joint extensor modulations in response to changes in the upward external force. Negative coefficients quantified the correspondence between joint flexor muscles and external force changes. Flex/Ext w coefficients ratio is a measure of the absolute relation between flexor and extensor joint muscles.

Apropos of point 1, if we assume that subjects’ biomechanical behavior was exclusively bound up with the upward force applied, we might expect similar muscular behavior (e.g., flexor and extensor *w* coefficients) for subjects interacting with similar external forces (e.g., PSE), arm posture being negligible different among subjects. On the contrary, inspections of the *w* coefficients distribution and Flex/Ext ratio of those three pairs of subjects with the most similar PSE values (Subj. 10, 2; Subj. 11, 12; Subj. 4, 5) show that it was not the case (Fig 9). In fact, both relative coefficients distribution and absolute Flex/Ext ratios were observed to be largely different despite of the very similar applied force (PSE), confuting the hypothesis that participants' muscular activity were just a direct consequence of the applied force. Moreover, the Flex/Ext ratios coefficients of all subjects do not show a significant correlation with respect to PSE individual values (*r* = -0.31, *p* = 0.27), indicating a not stable relationship between force applied (PSE) modulation and the pattern of muscular activity. Furthermore if the correlation we observed between perceptual decisions and muscular activity modulations was just protocol dependent, such a correlation should appear very high (or at least statistically significant) for each participants. On the contrary, as reported earlier, this was not the case for three subjects (subj.6, 7 and 9). Similarly, if arm muscular activity per se were constrained by the protocol to correlate with force modulation (perceptual decision), the correlation values in Fig 6B (i.e., individual PSE as function of Overall Muscular Activity) should be higher and more similar to that one describing the relation between psycho-metric and muscle-metric individual absolute thresholds (Fig 6C). Taken together these inspections support the hypothesis that despite of the relationship implied in our task between the variables considered, there is not an univocal correlation between the applied force and the modulation of the muscular activity supporting a pure biomechanical hypothesis. Instead we suggest that due to the high level of redundancy of the muscle-skeletal system, participants were able to complete the task with different (across subjects and trials) level of muscles co-activation that contributed partially to take the perceptual decision and consequently to drive the overall applied force distribution.

With regards to the second point, the biomechanical explanation hypothesis entails a high correspondences between *p*(‘YES’) and *p*(‘PoolEMG > criterion). Therefore, the attempt to predict *p*(‘YES’) by means of the muscle-metric curve would result in a very high goodness of fit for each subject. On the contrary our results do not fulfill completely these expectations. In fact we have shown through bootstrap simulations that subjects’ muscle-metric curves could not completely account for the perceptual decision variance but it explained at most around 60% (Fig 7). Likewise, we observed a larger and more asymmetric variance (above unity ratio) for slope ratio than the PSEs (Fig 8). This means that while comparing the two curves describing the muscles and perceptual decisions, they coincide fairly well in the absolute threshold whereas the psychophysical behavior is more variable than the muscular ones. Thus, if the muscle metric curve built on *p*(PoolEMG > criterion) would be just the description of a pure consequence of the force applied modulation, that in turn depends on the perceptual decision, the variance of the slope ratio between the two curves should be similar to the PSE ratio variance and symmetric around the slope ratio median. The lack of such an observation gives a clear indication that the muscle-metric curve could describe only partially the perceptual decision of our subjects, better in terms of its absolute threshold than in terms of its overall variability (i.e., precision).

As a whole we deem all our findings to be consistent proofs of the contribution of central signals in the sensation of force rather than an emerging, protocol dependent, effect.

### Muscular vs perceptual individual variability

We observed a wide variability among subjects sensitivity in detecting an upward force on the arm. Importantly, we showed that the inter-subject difference in psychophysical sensitivity was accurately predicted by the muscle-metric curves (Fig 6C). Moreover, we found that the individual PSEs variability correlated, for most of our subjects, with the variability of both individual joints torques and overall muscular activity (Fig 6A and 6B). This observation led us to hypothesize that the observed differences in the inter-subject perceptual performances is mainly linked with those processes of the perceptual decision involving the motor components of our perceptual task, rather than with ascending signals (e.g., cutaneous pressure on arm produced by the upward force). In fact, our subjects while judging the external force had to actively compensate gravity and counteract the upward force at the same time. Such a motor activity might have diminished (or “gated”) the perception of cutaneous stimuli by suppressing the transmission of afferent input (a process also called “*re-afference”*, [37]), as previously suggested by [38] for passive and active touch. Based on this assumption, in this study the force actively exerted by the arm was interpreted as a direct predictor of the final perception. We argue that because the muscular torque variables upon which the perceptual decision may hinge must counter two opposite forces acting on the arm (i.e., gravity and upward forces), their decoding by the CNS could be ambiguous and it could determine differences in detecting the lowest upward net force. Our findings support this interpretation by showing that in two cases PSE intensity were associated by either the residual or the net joint torque close to zero (Fig 6A), whereas in all the other cases this relation was not held. However it ought to be said that our conclusions drawn from the correlation observed between the elbow joint torques and subjective PSE have to be considered with caution. Since these variables are linked by a biomechanical constrain (i.e., the upward force applied have both a perceptual -PSE- and a dynamic -joint moment- correlate) the evidence of their correlation does not have an unique interpretation. Indeed the same correlation may be plausibly due to alternative decision-related explanations such as, for instance, subjective tendency to be more or less conservative to provide a positive answer, resulting in either a high or low PSE value respectively.

With regards to the muscular activity, during the perceptual task, the across subjects flexor muscles appeared to be more relevant than the extensors (Fig 5), the former being more strongly related with joint gravitational torque [39]. In fact, our participants tended to interact with the upward, anti-gravitational, forces more modulating the activity of the agonist flexor muscles rather than the antagonists involved to counteract the external force (i.e., extensors). Furthermore the muscles with the higher probability to compose the most predictive nested model (3 terms) were elbow and shoulder flexors (i.e., Bic and TrapU, see paragraph 1.7 in S1 Text), indicating that the muscles more related with the perceptual performance predictions were those ones likely involved in the compensation of the effects of gravity on the arm. It has been previously shown that gravity-related muscular effort is a good candidate to account for biases in haptic perception [40]. Similarly, a bulk of studies reported that judgments about force (i.e., weight and mass) are affected by changes in the gravitational torques acting on arm, as shown by microgravity experiments [1,2,41,42,43]. Also in everyday life, it is commonly experienced that, unless fatigued, moving and supporting our body in relation to gravity seem effortless and the muscular contraction involved to compensate gravity it is not constantly perceived [44]. Thus, we hypothesize that the participants’ modulation of gravity compensation entailed in our task might have influenced their judgments about the applied upward forces. In this respect, Sakajiri et al. [45] reported a perceptual bias while measuring the sensitivity to steering force exerted against or with gravity. As evidence of the biased interpretation of the gravity-related muscular effort, the authors observed a constant overestimation of the force exerted only for movements against gravity, since these entailed a supplemental muscle activity necessary to support the arm. Also, Lipshits et al. [46] cited in [47] asked participants to match a perceived downward force applied on the hand with an active upward hand torque. Although subjects performed the task remarkably well on Earth, when the same task was performed in microgravity conditions, they showed a consistent overestimation of the upward directed force, relative to an equivalent downward force. The authors interpreted higher upward forces as the result of CNS erroneous consideration of the gravitational torque acting on the arm also in 0g where, instead, it was absent. We suggest that similar effects as those reported above might explain the inter-individual differences observed here, where participants had to combine external with self-generated upward, anti-gravitational forces to maintain posture and judge the net force applied on their arm. Indeed we propose that the effects of gravity compensation observed in the present study can be associated with the concept of “voluntary negative motor command” used to explain involuntary contraction after-effects such as Kohnstamm’s manoeuvre (e.g., perception of arm lightness after deltoid long-lasting contraction) [48]. Within this neurophysiological framework the involuntary contraction—i.e., in our case self-generated gravity compensation—is inhibited by switching off the agonist muscles—i.e., flexors—that generate the involuntary action rather than by modulating the opposing antagonists. Interestingly, it has been speculated that both voluntary inhibition of involuntary actions (‘negative motor command’) and the drive of the involuntary action may converge in the motor cortex (as basal ganglia output, [49]), where the former does contribute an efference copy (providing conscious awareness) while the latter does not [48]. Therefore, similarly to the Kohnstamm’s phenomenon, we hypothesize that for some of our subjects, the sensory consequences of the constant counter gravity muscular activation could not be always accessible and so not perceived as self-generated but incorrectly attributed to the external upward force. Such an ambiguity between external and self-induced anti-gravitational force might be the source of variability we observed while perceiving the lowest level of upward force for some subjects whose PSE intensity did not coincided with a quasi-zero elbow torque.

Nevertheless, we cannot exclude the contribution of additional factors different from motor signals, that might have produced the spread of individual sensitivities, such as fatigue, fluctuations of attention and differences in decision criterion. Although only a carefully trial-by-trial analysis of the psychophysical behavior would permit to address this issue, the correlation found between individual psychophysical variability and individual motor activity lead to assign a lower contribution to other factors for the definition of the final perceptual performance. Further experiments are necessary to deeply investigate the weighting process of descending and ascending signals—as well as the influence of higher order cognitive processes- that central nervous system may carry out during force perception.

### Mapping muscle activity to force detection

The concordance between muscular and perceptual performance we observed is in agreement with the large body of works supporting the hypothesis that force perception is mediated by internal recognition of the descending motor command generating the muscular activity (i.e., sense of muscular effort). These studies involved force detection and discrimination protocols where motor output [2,7,8,13,14,17], afferent signals [11,12,15,16] and environmental conditions were manipulated [1,4,43,44,45,46,47]. In most of these investigations, authors used the muscular contraction of the contralateral limb where the stimulus force was applied as a measure of the perceived force (i.e., contralateral limb matching methods [16]). In the present study we showed that the analysis of the muscular activity associated to active force perception is an efficient and reliable predictor of the final perceptual decision.

Previous studies where arm EMG changes were recorded during motor task in both isometric and unconstrained conditions, have interpreted the increases or decreases in the magnitude of EMG traces as evidence of the changes in neural output [20,24,50,51,52]. On the other hand a bulk of works have been carried out to investigate the possible relation between perceptual decisions and sensory-driven changes in neural activity ([53] for a review). In accordance with these trends of investigations and with the aim of providing insights into the brain mechanisms underpinning force perception, in the present work we described the perceptual decision about force by modelling the changes in muscular activity interpreted as changes in α-motoneuron output. Indeed, the changes observed in the PoolEmg and the trend of the upward forces correlated significantly for 85% of our subjects. For this group of participants we showed that their global arm muscular activity is able to explain around 60% of the total variation of their psychophysical performance, being this a measure of the contribution of the central processes involved in our detection task. Interestingly such a muscular predictive power appeared to be relatively stable across different muscle pooling (nested models) where the number of predictive muscles were reduced (Figs 7 and 8). The outcomes of our simulations suggest that, in our task, muscles were efficiently co-activated to maximize relevant information about the external force. Further investigations are needed to explore what pattern of muscles synergies, among subjects, provide highest predictive power about force perception.

We showed that 2 out of 14 subjects presented a very low muscle-psychophysics concordance. A possible explanation can be that those participants, while detecting upward forces, mostly modulated other muscles with vertical pulling directions—such as Pectoralis Major and Lateral Deltoid—that were not recorded and that probably described their global muscular activity at best.

To a neuro-metric framework, the level of similarity that we observed between muscles (interpreted as neural output) and subjects performance is in accordance with previous works linking the sensitivity of cortical neurons with animals visual perception in discrimination and detection task [54,55,56,57]. These authors summarized the relationship between neural and behavioral sensitivity by computing the ratio of neural/psychophysical thresholds. The reported geometrical means of the threshold ratios they observed, ranged from 0.87 to 1.51 [56]. On the other hand the similarity in precision was expressed as slope ratio whose geometrical means ranged from 0.99 to 1.16. Comparable slope and threshold ratio were found in the present study between the muscles and behavior. In fact the median across subjects of the threshold ratios geometric mean, ranged from 0.94 to 0.97 for all nested models while the range of median values of slope ratios was 0.73 to 1.3. Specifically the consistent presence of above unity slope ratios (Fig 8) values indicates a relevant difference between the variability of the muscles versus the psychophysical performance, with higher variation for the latter. The resultant reduced precision observed for perceptual decisions is not surprising given its higher level of elaboration with respect to muscles activation.

Finally from a neurophysiological point of view, previous studies using TMS were carried out to assess the modulations of corticospinal excitability in observer’s motor system during the observation of lifting objects with different weights. In particular, some of these studies demonstrated that neurons in M1 are able to simulate observed weight-lifting actions in terms of detailed motor process [58,59]. More interestingly, Alaerts et al. [19] who used TMS perturbation while subjects observed two different lifting actions, was able to show that M1 can encode the muscle requirements for the lifting action not only in terms of muscle involved but even in the terms of force that is produced by the particular muscles. Moreover, these authors, that did not observed statistical differences in corticospinal excitability when the observed actions involved small change in the force exerted, suggested that M1 force encoding is expected to be more accurate during the actual execution and when relatively larger forces (> 2kg) were applied [19]. Since these suggested conditions characterized our task, we believe that our quantification of the predictive power of the EMG modulations about the psychophysical performances might be linked with the corticospinal signals characterizing quasi-isometric force perception.

## Supporting Information

S1 DataReadme Description of the data structures organized and stored in four compressed archives. (PDF)

S1 DataSet Compressed archive containing four Matlab data structures of all relevant data presented in the manuscript. (ZIP)

S2 DataSet Data structure (*Emg_subjs_1to5*.*mat*) containing EMG data of subjects 1, 2, 3, 4 and 5. (ZIP)

S3 DataSet Data structure (*Emg_subjs_6to10*.*mat*) containing EMG data of subjects 6, 7, 8, 9 and 10. (ZIP)

S4 DataSet Data structure (*Emg_subjs_11to14*.*mat*) containing EMG data of subjects 11, 12, 13 and 14. (ZIP)

S1 Text File containing the supporting information and supporting figures cited in the manuscript. (DOCX)
