# Supplementary material for: Mapping Muscles Activation to Force Perception during Unloading
Source: PLoS One. 2016 Mar 31;11(3):e0152552. doi: 10.1371/journal.pone.0152552 (PMC4816335; doi:10.1371/journal.pone.0152552)
Supplement: S1 DataReadme — (PDF) [file pone.0152552.s001.pdf]

Instructions for DataSets Interpretation

The Matlab files attached contain the data structures used in the analysis presented in the manuscript:

Emg_subjs_1to5/6to10/11to14; All_psyEmg; **All_MultiReg**; **All_Torques**; **All_simuOutput**

For all data sets *n* is a specific subject and *tr* represents the single trial.

Emg_subjs_1to5/6to10/11to14(n).mat (in S2, S3, S4_DataSet.zip, respectively) contains subjects data about the EMG signals already rectified and filtered as described in the manuscript. All the analysis and figures referring to muscles activity were obtained from these data (e.g., MAV, PooleEmg).
The structure is organized according to this organizational chart:

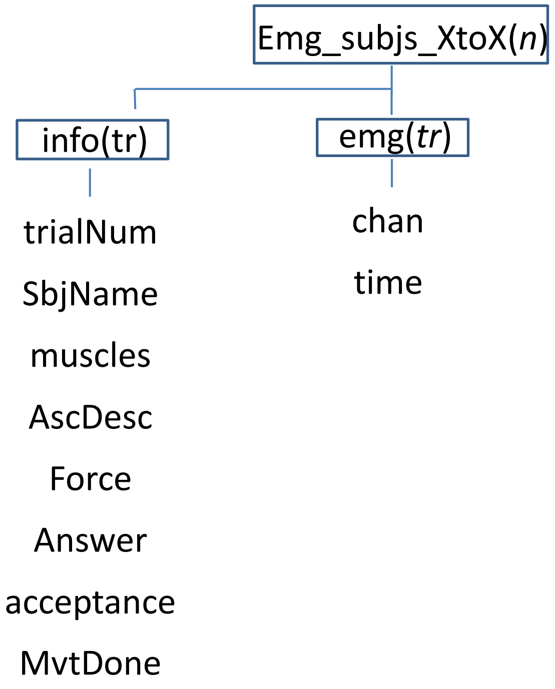

info(tr).trialNum, info(tr).SbjName and info(tr).muscles report the trail considered, subject's name and muscles names order as stored in *emg(tr).chan*.

info(tr).AscDesc= 0 represents ascendant staircase force stimuli presentation while 1 descendent staircase. *info(tr).Force* is the intensity (in Newton) of the upward force presented at trial *tr*. *info(tr).Answer*= 0 indicates 'NO' subject response while 1 stays for 'YES'. *info(tr).acceptance*= 0 indicates trial excluded because of emg artifacts, 1 is for trial accepted for the analysis. *info(tr).MvtDone*= 0 indicates either whether endpoint did not exceed target limits or arm joints configuration did not differ from initial arm posture, 1 otherwise.

emg(tr).chan contains the EMG signal values of all eight muscles (rows) for trial *tr*. Thus the order of muscles name in *info(tr).muscles* corresponds to the number of rows in *emg(tr).chan. emg(tr).time* is the time vector of the EMG signal for the same trial.

All_psyEmg(n).mat contains subjects data for the analysis performed to obtained psycho and muscle-metric curves and associated parameters. With this data structure Figure 2,3 as well as tables 1 and 2 can be reproduced.
Per each trial *tr type*= 0 is for ascendant staircase and 1 is for descendant. *EmgResp* is the PoolEMG obtained as described in the manuscript. *PSEstair* is the absolute threshold extracted from staircase method as described in the manuscript. *PSEci* is the 95% confidence intervals. *Pyes* is the extracted probability of answer 'YES' given each upward force level. *upForces* is the vector of forces presented (with 0 and 30 as minimum and maximum values) without repetitions and excluding unaccepted trials. *Alphs* is the

intercept parameter obtained from the GLMfit (Matlab Statistics Toolbox) of the psychophysical answers as described in the manuscript, *Beta* represents the slope parameter. *CurveFit* and *PointFit* are the y and x axis values, respectively to draw the same psychometric curves presented in figure 2. *Sigma, PSEfit* and *PSEfitCI* are the absolute thresholds, its standard deviation and 95% confidence intervals, respectively, obtained by the psychophysical fit. *Rsqr* is the goodness of the logistic fit obtained from the psychophysical data. *Pdw* represents the probabilities of the PoolEMG to be higher than its mean for each forces presented. *EmgUpForces* is the vector of force level presented excluding unaccepted trials. *EmgAlpha, EmgBeta, CurveFitEmg, PointFitEmg, SigmaEmg, PSEemg* and *RsqrEmg* have the same nomenclature used for the psychophysical data but they refer to muscle-metric fit. *RsqrtTot* is the goodness of the logistic fit obtained by merging psychophysical and muscular probability together. *FitCmpPsy* and *FitCmpEmg* are the outputs of the Matlab Two-Sample Kolmogorv Smirnof test performed between the psychophysical and the muscular response distribution with respect to the distribution of the two datasets merged.

All_MultiReg(n).mat contains subjects data concerning the muscular multiple regression output (e.g., standardized regression coefficient per each muscle). With this data structure Figure 4 can be reproduced. *Correlcofs* reports the correlation coefficients of the simple regression performed for each pair of muscles. *ChanName* reports the muscle name of each EMG channel. *Outputs* is a structure containing the multiple regression intercept and coefficients of each muscle (*Outputs.coef*) while *Outputs.cInt* and *Outputs.stati* are the confidence intervals and the statistics of the obtained regression coefficients.

All_Torques(n).mat contains the data concerning joint shoulder and elbow flexion torques (total and residual) of those ten subjects from which joints torque were calculated. With this data structure Figure 5A can be reproduced.
shFlex(tr) and *elbFlex(tr)* are structures storing total -*.total*- and residual -*.residual*- shoulder and elbow flexion torques profiles, respectively, for trial *tr. Ttime(tr)* is the associated time vector and *Tupforce(tr)* is the force level presented at trial *tr*.

All_simOutput(n).mat contains subjects data obtained from the simulations (1000 bootstrap resampling) described in the manuscript. With this data structure Figure 7,S3,S4 and S5 can be reproduced. This structure contains a sub-structure named *boot(k)* where *k* represents each nested muscular model obtained as described in the manuscript.
In *All_simu(n).boot(k) resmpPool, resmpForc* and *resmpAns* are the resampled distribution of the PoolEmg, the resampled force level presentation order and the resampled psychophysical answers order, respectively. *Thres, Inter* and *Slp* are the resultant psychophysics parameters extracted from these distributions while *medianThres, medianInter, medianSlp, SEthres, SEinter, SEslp,CIThres, CIinter* and *CIslp* are the associated descriptive statistics. The same nomenclature was adopted for the parameters and statistics obtained by muscular data. *pvalDist* is the p-values obtained by submitting a Two-Sample Kolmogorv Smirnof to test the statistical difference between the psychophysics and the muscular activity distribution. *Rsquare* contains the goodness of fit extracted to quantify how much muscle-metric curve accounts for the probability of answer 'YES' while *RsqMedian* and *RsqSe* are the relative descriptive statistics. Finally *IntercCE*, *SlopeCE* and *ThresCE* contain respectively the differences between psycho-metric and muscle-metric parameters values.
