# Supplementary material for: Mapping Muscles Activation to Force Perception during Unloading
Source: PLoS One. 2016 Mar 31;11(3):e0152552. doi: 10.1371/journal.pone.0152552 (PMC4816335; doi:10.1371/journal.pone.0152552)
Supplement: S1 Text — (DOCX) [file pone.0152552.s006.docx]

*Title:* **Mapping Muscles Activation to Force Perception During Unloading**

**Supporting Information**

**S1 text**

- 1. **Apparatus details**

The relation between the counterweight on the Track-Hold balancer (Cw) and the resultant upward force *F* acting on subject’s arm is given by the following equations:

$$Cw\cdot\overline{O_{1}O_{3}^{'}} \cdot\sin\left( \alpha\right)=F\cdot\overline{O_{1}O_{3}}\cdot sin(\alpha)$$

from which:

$$\frac{\overline{O_{1}O_{3}}}{\overline{O_{1}O_{3}^{'}}}=\frac{Cw}{F}= 3$$

where $\overline{O_{1}O_{3}^{'}}$ is the distance between the pivot point (*O1*) of the rigid link attached to the basis and the load application point, $\overline{O_{1}O_{3}}$ is the distance between the pivot point of the rigid link attached to the basis and the point of upward force exertion where sin(α) describes the elevation angle of each device configuration (Fig. 1, main text). Therefore for the geometric properties of the mechanism that keeps the ratio ${\overline{O_{1}O_{3}}}/{\overline{O_{1}O_{3}^{'}}}$ constant, a downward forces Cw applied to the balancer produces always one third of the same force *F* in the upward direction at point *O*3, that is constant at any configuration of the device. On the other hand, the point of force application (i.e., *Xf*) on subjects arm was determined by the following equation:

$$X_{f}=\frac{(1+\frac{L2}{L1} )}{\left( 1+\frac{P_{UA}}{P_{D}} \right)+\frac{L2}{L1}} \cdot L3$$

where *L*1 is the distance between the shoulder joint and the upper arm center of mass, *L*2 is the distance between the upper arm center of mass and the elbow joint and *L*3 is the distance between the elbow and the center of mass of the segment formed by the forearm and the hand. *Pua* and *PD* represents the upper arm weight and the sum of the weights of the forearm and the hand, respectively. Interestingly by applying a single constant and vertical force on *Xf* it is possible to reduce the elbow and shoulder articulation couples necessary to balance the proper weight of the whole arm [1]. The segment length of upper arm, forearm and hand were directly measured from participants’ limb. The center of mass and the weight of each upper limb segment were estimated by means of [2] and [3].

- 1. **Visual Feedback Details**

Two 3D spheres were projected on the frontal panel of a virtual reality system (*VRs*) composed of four projection panels, four 3D projectors (1024 x 768 pixels, 60 Hz) and shutter glasses providing stereoscopy. A custom XVR (VR Media, S.r.l.) routine transformed the time varying *Xf* coordinates obtained from Track-Hold (TH) magnetic sensors to VRs coordinates. Moreover, the new roto-translated point was shifted away from subjects’ body along the z axis, transverse plane, of an amount that corresponded to the actual distance between *Xf* and subject’s hand palm. This procedure allowed to update (60 Hz sampling rate) the cursor sphere position that corresponded to subject’s hand in the virtual scene.

- 1. **Kinematic and Kinetic Model of the Arm**

Joint angles *q*, joint velocities $\dot{q}$ and accelerations $\ddot{q}$ were used to estimate the torque profiles actively generated by the subjects, via recursive Newton-Euler calculation (*rne* function of Matlab Robotics toolbox Corke, [4,5]). Total torque, **τ** , was computed as follow

$$\boldsymbol{\tau}=M\left( q \right)\ddot{q}+C\left( q,\dot{q} \right)\dot{q}+G(q)$$

where *M* is the matrix of principal inertia moments, *C* is the Coriolis and centripetal torque and G is the gravitational torque. The non-gravitational (residual) torque was extracted by subtracting gravitational torque from the total torque. In other words, residual torques describe the portion of the active muscular moment exerted at arm joints taking into account only the external upward force applied.

As described in Russo et al. 2014 cited in the manuscript, the model defined a chain of articulated links through Matlab Robotics toolbox describing the position and orientation of Cartesian reference frame fixed on each link with respect to the reference frame fixed on the preceding link. The rotation axis of each joint being the z axis of the preceding link in the chain. In accordance with D-H convention the x axis in each frame is directed as the normal between the rotation axis of that frame and the rotation axis of the next frame (see Russo et al., 2014 cited in the manuscript for details on the homogenous transformation matrix). For each time sample and each joint angle, a vector between two markers aligned with the axis of the limb segment defining the rotation of that joint was computed first (i.e., shoulder and elbow markers for shoulder adduction and shoulder flexion, elbow and wrist markers for shoulder external rotation and elbow flexion). Then the associated angle was computed for each defined limb segment as $\tan^{-1} (y/x)$, where x and y are the coordinate of the vector in the reference frame associated to the joint rotation axis z (Russo et al., 2014). A sketch of the definition of the above mentioned joint angles (with exception of shoulder external rotation not considered in the manuscript) is presented in Figure A, where $\theta_{shoFlex}$ and $\theta_{elbFlex}$ are the shoulder and elbow flexion angles, respectively, whereas $\Psi_{shoAdd}$ is the shoulder adduction angle. ${\boldsymbol{net} \boldsymbol{\tau}}_{shoFlex}$ and ${\boldsymbol{net} \boldsymbol{\tau}}_{elbFelx}$ represent the total flexion torque generated by the subject at the shoulder and elbow joint respectively. ${\boldsymbol{net} \boldsymbol{\tau}}_{shoAddu}$ describes the total adduction torque generated by the subject around shoulder joint. $\boldsymbol{\tau}_{ext}$ and $\boldsymbol{\tau}_{g}$ are the moment of the upward external force and gravitational forces, respectively, acting on joints arm. Moments produced by the upward external force and the gravitational forces have opposite signs, therefore all moments actively generated by the participants to counteract gravity will have positive sign, while joint moments aimed to resist upward external forces will be negative.

Our dynamic model of the arm and the model previously used by Russo et al. [6] slightly differed for two reasons. Firstly, we estimated centers of mass and the weight of each upper limb segment by means of Zatsiorsky et al. equations [2] and de Leva adjustments [3] from direct measurements of individual length as well as girth of the upper arm, forearm, and hand. Secondly, we introduced in the recursive Newtown-Euler calculations of torques an external force (with its relative force momentum) representing TH upward force acting at point *Xf* of subjects’ arm at each trial (*rne_dh* function). This allows us to obtain, for each upward force presented, both shoulder and elbow time varying joints flexion torques. Since we required a quasi-isometric task, as long as subjects remained in the target the torque measures were approximately constant. Thus, the median and the standard error of both shoulder and elbow joint torque vectors were extracted for each trial.

- 1. **Criterion Selection**

Couched in neuro-metric terms, the decision about the stimulus force that was required in the present experiment can be generally described as a two steps process: the first concerning the brain encoding of the relevant features of the sensory stimulus, that in our case it is assumed to occur, in addition to the elaboration of afferent signals, through a representation of the motor commands sent to arm muscles related to each external force level (central mediated hypothesis of force perception), while the second entailing the interpretation of these motor signals to drive a perceptual decision [7]. The latter, called the read out process, is the transformation of the signals representing the stimulus strength into a distribution of specific trial-by-trial “behaviors” (i.e., decision variable), namely psychophysical choices and muscle pattern activations above a criterion level [8,9]. In the present study the decision variable was described by the probability function of our quantification of the overall muscular activity (i.e., PoolEmg). As reported in the text such a decision variable allowed us to describe the arm muscles activity in terms of exerted muscular force: high and low PoolEmg representing either downward (to resist the external upward force), extensor or upward (to counteract gravity on arm), flexor activity, respectively. The former and the latter extremes of this continuum being related to the highest probability to detect and to not detect the upward force, respectively. Thus, the muscle-metric curve could be constructed by assuming that muscles “detect” the upward force every time that a stimulus level is associated to a PoolEMG activity (i.e., decision variable) over a threshold (i.e., criterion). In the present study the selection of such a threshold was carried out by first assigning to the criterion different values within the decision variable distribution (i.e., mean value, ± 1 and 2 standard deviation from the mean) and then, by looking at what values provided the best concordance between the psychophysical and muscle-metric curves. Figure B reports the outcomes of this inspection for two typical subjects (subj. 1 and 11) whose criterion values were set to the individual mean of the PoolEMG distribution and ±1 SD from the mean. It is readily visible that the muscle-metric curve constructed with the criterion set to the mean of the PoolEMG resembles at best psychometric curve in terms of threshold and shape.

- 1. **Bayesian Information Criterion**

An outline of the BIC (in this study called BaIC to differentiate it from biceps nomenclature) was originally given by Schwarz [10] who provided a criterion for model selection based on Bayesian theory. The aim of Bayesian model selection is to obtain the posterior probability of model *M* given the data *y*, *p*(M|y). As reported in [11], Bayes’ theorem allows to calculate this posterior probability as:

$$p(M|y)=\frac{p\left( y | M \right)*p(M)}{p(y)}$$

With *p*(*M*) being the prior probability of the model, *p*(*y*) the probability of the data and *p*(*y|M*) the likelihood of my data given the model. Since the latter depends on the selection of the model parameters values, likelihood results a complicated procedure for most models [11]. Nevertheless, it has demonstrated that it is possible to approximate full-scale Bayesian model selection by correcting the maximized log likelihood and obtaining the Bayesian Information Criterion, BIC [10,12] with the following formula:

$$BIC=-2\ln L\left( \theta| y, M \right)+K lnN$$

where *-2 Ln L* (*L* being the likelihood of our data given the model parameters *θ*) is the deviance of the nested model, *N* is the number of data points on which the likelihood calculation is based - in our case the number of trials per regression term- and *K* is the number of model parameters (here the multiple regression slope and intercept). Therefore, looking at the addition terms it can be noticed that the nested model with the best posterior probability given the data will be represented by the BIC closest to zero, namely that nested model providing the lowest deviance (in the present study the highest likelihood to yield the empirical PoolEMG data) and being composed by the lowest number of terms *N* (i.e., highest simplicity). Nevertheless, although BIC measure is an effective tool for models comparison when simplicity must be maximized, the weight assigned to simplicity could partially mask the actual deviance when *N* presents wide changes among models as it occurs in the present study. For instance, in our case, the observation of relevant reduction between BIC values of the general and the least (one term) model might be due just to the *N* reduction rather than to an actual deviance decrease. In the light of this possible BIC ambiguity, as reported in the main text, we performed model comparisons by considering a trade-off between BIC values and the amount of the explained PoolEMG variance (R² PoolEmg in the main text) provided by each nested model. The latter measure was defined as 1-SSE/SST, with SSE being the sum of square residuals of the data predicted by the nested model with respect to the empirical PoolEMG data, and the SST the sum of the squared residuals of the empirical data with respect to its mean.

**1.6 Simulations Output**

Within each group of likelihoods, the best, 3 terms, and the least, 1 term, models appeared to be those with higher likelihood, with the latter showing a likelihood always higher than the former (N. of Predictors = 1 in the first three plots from the top plots of Figure D). Nevertheless it ought to be noted that although model with only one predictor is always the more likely to provide the highest account of perceptual performances, its range of *R²* values are also the most variable. In fact, as shown in the lowest plot of Figure D the inverse of the coefficient of variation (i.e., CV, calculated as the ratio between the mean and the standard deviation of the R² distributions) associated to the 1 predictor model is more than the double with respect to the other nested and general models measures. In other words the nested muscular model with just one term is as likely to have the highest predictive power as to provide the lowest level of predictability with respect to all other models.

**1.7 Muscles probability for best predictive nested model**

Since subjects had different muscles composing each nested model, we extracted the probability of each muscle to compose the BEST, three terms, model. We found that across subjects and simulations, Bic and TrapU were the muscles with a probability higher than 50% to compose the three terms muscular model, namely 86% and 64%, respectively. These two muscles were those presenting highest values of *w* coefficients (Fig. 5, main text). Two other muscles that showed a relatively high probability were DeltA and LatD, with a similar probability of 43% to compose the three terms model. Interestingly three muscles out of the four mentioned above are joint flexor (Bic, TrapU and DeltA) while, only one is a joint extensor (LatD). Such an observation indicates that the muscles that are more likely associated with the perceptual performance predictions are not those that counteract the external upward forces (extensors), rather those that reduced their contraction with respect to force level increase (flexor).

**References**

1. Lenzo B, Fontana M, Marcheschi S, Salsedo F, Frisoli A, Bergamasco M. Trackhold: a novel passive arm-support device. Asme. 2015 Jan 30; doi: 10.11151/1.4031716.

2. Zatsiorsky VM, Seluyanov VN, Chugunova LG. Methods of determining mass-inertial characteristics of human body segments. In Contemporary Problems of Biomechanics. Chernyi G.G. and Regirer S.A. CRC Press, Massachusetts. 1990: 272-291.

3. de Leva P. Adjustments to Zatsiorsky-Seleuyanov's segments inertia parameters. J. Biomechanics. 1996; 29:1223-1230.

1. Corke PI. A robotics toolbox for matlab. IEEE Robot Autom. 2011 Mag 3; 24-32. doi: 10.1109/100.486658
2. Corke PI. Robotics, vision and control. Berlin Heidelberg: Springer-Verlag. doi: 10.1007/978-3-642-20144-8
3. Russo M, D’Andola M, Portone A, Laquaniti F, d’Avella A. Dimensionality of joint torques and muscle patterns for reaching. Front Comput Neurosci. 2014 Mar 3; 8:24. doi: 10.3389/fncom.2014.00024.
4. Gold JI, Ding L. How mechanisms of perceptual decision-making affect the psychometric function. Prog Neurobiol. 2013; 103:98-114.
5. Swets JA. The relative operating characteristic in psychology. Science. 1973; 182:990-1000.
6. Parker JI, Newsome WT. Annu Rev Neurosci. 1998; 21:227-277.
7. Schwarz G. Estimating the dimension of a model. Ann Stats. 1978; 6:461-464.
8. Lewandowsky S, Farrel S. Computational modeling in cognition: principles and practice. 1^st^ ed. Thousand Oaks, California: Sage; 2011.
9. Khua J. AIC and BIC: comparisons of assumptions and performance. Sociological Methods & Research. 2004; 33:188-229.

**Figure A**

**
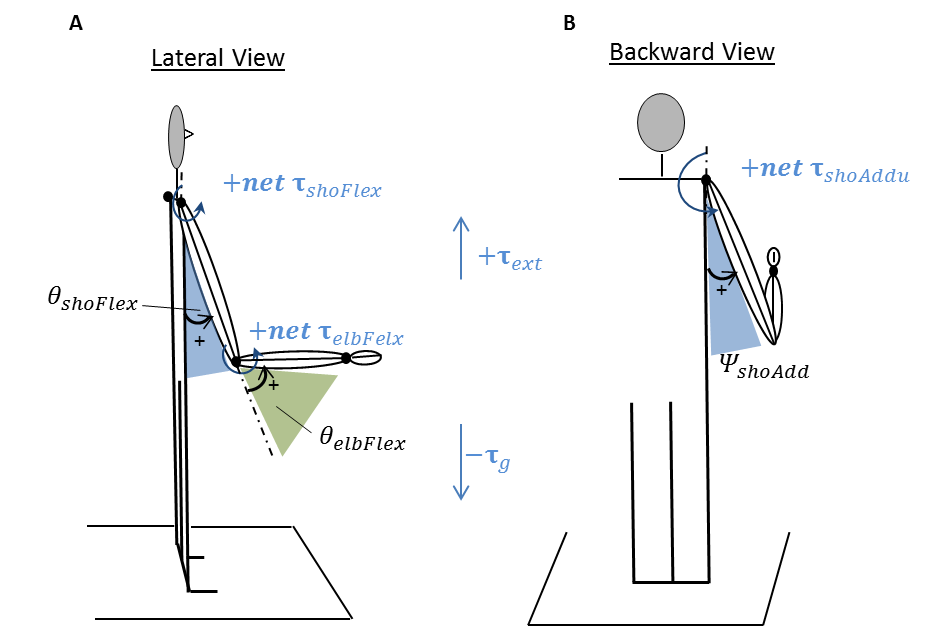
**

**Figure B**


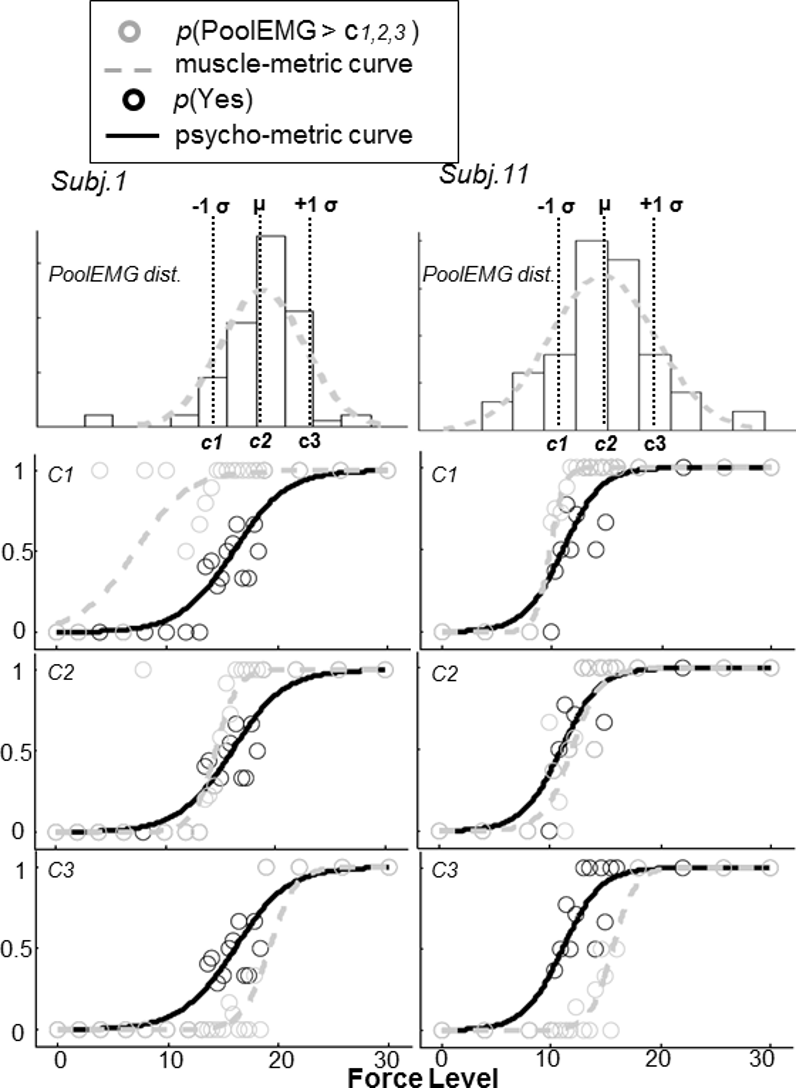


**Figure C**

**
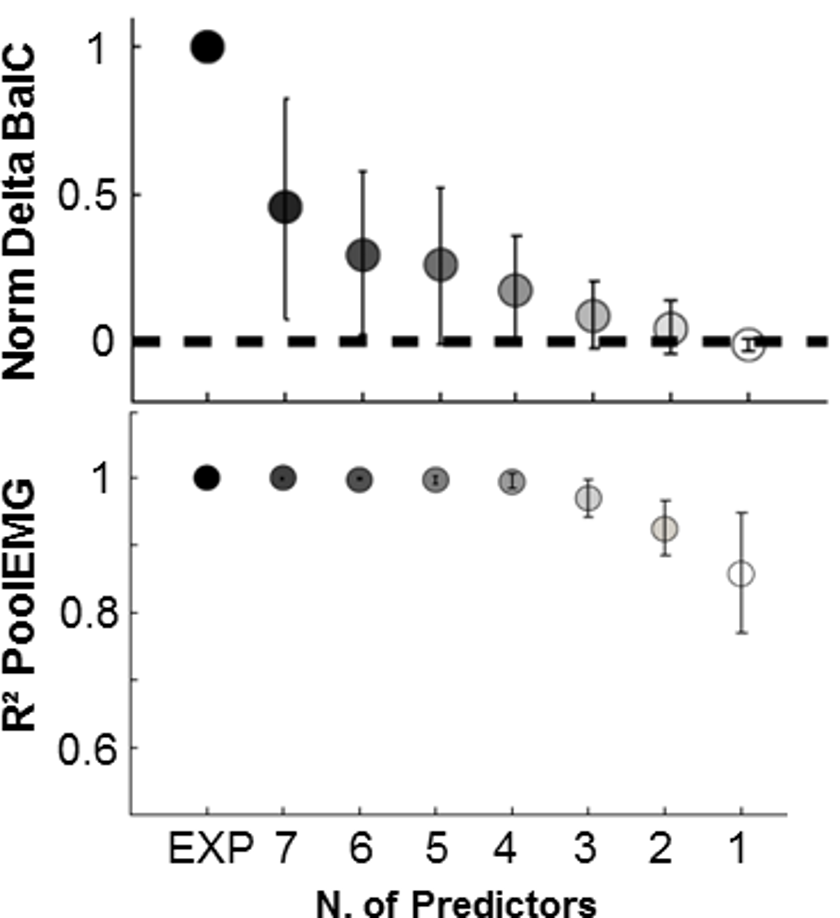
**

**Figure D**

**
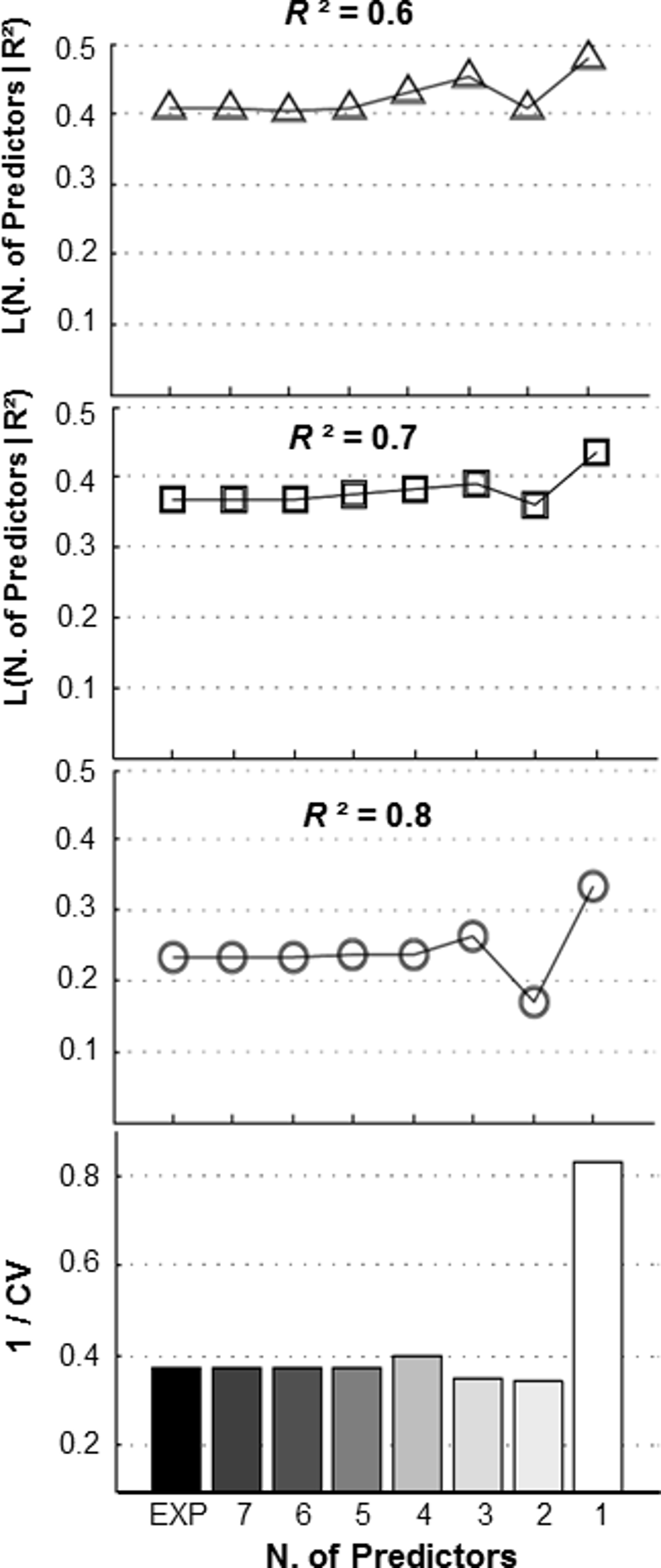
**

**Figures Legend**

**Figure A: Sketch of joints angles definition.**

$\theta_{shoFlex}$ and $\theta_{elbFlex}$ are the shoulder and elbow flexion angles, respectively, whereas $\Psi_{shoAdd}$ is the shoulder adduction angle. ${\boldsymbol{net} \boldsymbol{\tau}}_{shoFlex}$ and ${\boldsymbol{net} \boldsymbol{\tau}}_{elbFelx}$ represent the total flexion torque generated by the subject at the shoulder and elbow joint respectively. ${\boldsymbol{net} \boldsymbol{\tau}}_{shoAddu}$ describes the total adduction torque generated by the subject around shoulder joint. $\boldsymbol{\tau}_{ext}$ and $\boldsymbol{\tau}_{g}$ are the moment of the upward external force and gravitational forces, respectively, acting on joints arm.

**Figure B: Criterion Selection.**

Left and right columns represent PoolEMG distribution as well as muscle-metric (gray) and psychometric (black) curves (lines) and probabilities (dots) of 2 subjects. *Upper histograms* c2, c1 and c3 represent PoolEMG mean, -1σ below and 1σ above the mean, respectively. *Upper curves* depict the resultant probabilities and fitting curves when criterion was set to c1, i.e., *p*(PoolEMG > -1σ ). *Middle curves* Resultant probabilities and fitting curves with criterion set to c2, i.e., *p*(PoolEMG > µ ). *Lower curves* Resultant probabilities and fitting curves with criterion set to c3, i.e., *p*(PoolEMG > 1σ)*.*

**Figure C: Statistics for BEST Nested Model Selection.**

*Upper graph* reports normalized Delta BaIC averaged across subjects ± SD, obtained by the backward eliminations procedure, as measure of each model trade-off between its likelihood in providing the empirical PoolEMG data and its simplicity. *Lower graph* depicts model comparison statistics (median ± SE across subjects) obtained by the backward eliminations procedure, where R² PoolEMG values quantify how much each nested model is able to predict the empirical PoolEMG activity. In both graph the model with 8 predictors (i.e., *EXP*) represents the empirical data.

**Figure D: Likelihood of each nested model given a target *R².***

*Upper graphs* Likelihood values of each nested model (N. of Predictors) given a specific amount of explained variance (*R²*). Likelihood measures were extracted from the *R²* distribution curves depicted in Figure 7. *Lowest* *bar graph* represents the inverse of the coefficient of variation (i.e., CV) calculated as the ratio between the mean and the standard deviation of the R² distributions depicted in the main plot. Thus, the lower bars, the lower spread R² distribution.
